# Enriched Oxygen Vacancy‐Mediated Efficient Charge Transfer in S‐Scheme ZnO‐V_O_@Zn_0.5_Cd_0.5_S Heterojunction for Rapid U(VI) Removal From Nuclear Wastewater

**DOI:** 10.1002/advs.202512163

**Published:** 2025-09-12

**Authors:** Cailing Liu, Xiaohui Ren, Smruti Ranjan Sahoo, Artem Kuklin, Chen Yao, Hans Ågren, Ye Zhang

**Affiliations:** ^1^ School of Resources Environment and Safety Engineering University of South China Hengyang 421001 P. R. China; ^2^ Lab of Optoelectronic Technology for Low‐Dimensional Nanomaterials School of Chemistry and Chemical Engineering University of South China Hengyang 421001 P. R. China; ^3^ The State Key Laboratory of Refractories and Metallurgy Key Laboratory for Ferrous Metallurgy and Resources Utilization of Ministry of Education & Hubei Provincial Key Laboratory for New Processes of Ironmaking and Steel making Faculty of Materials Wuhan University of Science and Technology Wuhan 430081 P. R. China; ^4^ Department of Physics and Astronomy Uppsala University Box 516 Uppsala SE‐751 20 Sweden; ^5^ Faculty of Chemistry Wroclaw University of Science and Technology Wyb. Wyspianskiego 27 Wroclaw PL‐50370 Poland

**Keywords:** nuclear wastewater treatment, oxygen vacancies, photocatalysis, rapid U(VI) removal, S‐scheme heterojunction

## Abstract

Efficient charge separation is critical for high‐performance photocatalytic reduction of U(VI) in nuclear wastewater. Employing defect engineering, an oxygen vacancy (V_O_)‐enriched S‐scheme ZnO‐V_O_@Zn_0.5_Cd_0.5_S (ZnO‐V_O_@ZCS) heterojunction is constructed for rapid U(VI) removal. The synergistic effect of oxygen vacancies and the S‐scheme mechanism is shown to significantly enhance charge separation and photocatalytic U(VI) reduction. Remarkably, ZnO‐V_O_@ZCS achieves 99.10% U(VI) removal within 10 min at pH 4 under simulated sunlight without sacrificial agents, surpassing the V_O_‐free ZnO@ZCS reference and exhibiting 4.10‐fold and 20.0‐fold higher activity than pristine ZnO‐V_O_ and ZCS, respectively. Meanwhile, the catalyst maintains robust performance across a wide pH range (3–8), complex matrices (interfering ions/dyes), natural sunlight, and diverse U(VI) containing wastewater. In situ X‐ray photoelectron spectroscopy (In situ XPS) and Kelvin probe force microscopy (KPFM) confirm the S‐scheme charge transfer between ZnO‐V_O_ and ZCS. Femtosecond transient absorption spectroscopy (fs‐TAS) and density functional theory (DFT) calculations reveal that V_O_ serve as transient electron traps, creating rapid charge‐transfer channels that interplay with the S‐scheme to enhance charge transfer efficiency and accelerate U(VI) reduction kinetics. This study provides new insights for designing defect‐modulated S‐scheme heterojunction photocatalysts, promising for sustainable nuclear wastewater treatment.

## Graphical abstract

An S‐scheme ZnO‐V_O_@Zn_0.5_Cd_0.5_S heterojunction with oxygen vacancies (V_O_) was developed for rapidly removing U(VI) from nuclear wastewater. The results show that the synergy between S‐scheme transfer and V_O_ enhances the catalyst’s U(VI) removal kinetics. It achieves 99.10% removal in 10 minutes without any sacrificial agents. Furthermore, it presents excellent U(VI) removal performance in diverse complex scenarios, enabling sustainable uranium recovery.

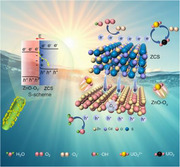

## 1 Introduction

As a clean and efficient energy source, nuclear energy holds an irreplaceable position in modern energy systems.^[^ ^1^ ^]^ Radionuclide uranium (U) is a crucial element for the long‐term sustainable development of nuclear energy, and generates a huge demand for U in the future.^[^ ^2^, ^3^ ^]^ Nevertheless, uranium mining wastewater poses critical environmental risks due to the presence of highly mobile, radioactive, and toxic soluble U(VI) species.^[^ ^4^, ^5^, ^6^ ^]^ Compounding this challenge, such wastewater frequently contains coexisting U(VI), organic compounds, and diverse anions/cations, which significantly hinder effective U(VI) removal. To address these issues, solar‐driven photocatalytic reduction technology has emerged as a targeted strategy, enabling the conversion of soluble U(VI) to insoluble U(IV) for effective U contamination remediation.^[^ ^7^ ^]^ The technology combines the advantages of environmental friendliness, economic feasibility, and high selectivity, providing a practical solution for the green transformation of the nuclear energy industry and long‐term ecosystem protection.^[^ ^8^, ^9^, ^10^ ^]^

Developing efficient and stable photocatalysts is a key to realizing efficient photocatalytic reduction of U(VI). Although various photocatalytic materials have been developed for U(VI) removal, most of them require a long period to reach a high removal efficiency, primarily attributed to inherent material defects, including low photogenerated carrier migration rate and fast surface complexation rate.^[^ ^11^ ^]^ The emerging S‐scheme heterojunction provides a new strategy to solve this problem. In this system, the Fermi energy level (*E*_f_) difference between two semiconductors drives electrons to spontaneously migrate from the semiconductor with the higher *E*_f_ to the one with a lower *E*_f_, thereby creating a strong internal electric field (IEF).^[^ ^12^, ^13^ ^]^ The Coulomb force generated by the IEF acts to promote a rapid complexation of weak redox carriers while retaining high redox potential carriers under the exposure of light, thus enhancing the charge separation efficiency while maintaining the strong redox capacity and enhanced photo‐corrosion resistance.^[^ ^13^, ^14^, ^15^ ^]^ In addition, further optimization of the S‐scheme heterojunction through the precise design of the interfacial vacancy structure can achieve multiple synergistic enhancement mechanisms such as the broadening of the light absorption spectral range, the construction of electron transport bridges, and the increase in the density of surface active sites. For example, Zu et al.^[^ ^16^ ^]^ designed a tunable WO_3_‐O_V_/In_2_S_3_ S‐scheme heterojunction, in which oxygen vacancies not only broadened the light absorption range of the material but also acted as an electron transport channel at the heterojunction interface to synergistically enhance the thermodynamic and kinetic properties of the catalytic reaction. Furthermore, the S‐vacancy (V_S_) modified In_2_S_3_/In_2_O_3_ heterojunction developed by Lai et al.^[^ ^17^ ^]^ precisely modulated the electron density distribution and active site density on the surface, which significantly enhanced the CO_2_ adsorption and activation efficiencies, and realized the highly efficient photocatalytic CO_2_ reduction.

Zinc oxide (ZnO) has attracted considerable research interest owing to its adjustable bandgap, inherent non‐toxicity, and exceptional cost‐effectiveness, while its practical implementation remains constrained by a narrow optical absorption spectrum and rapid charge carrier recombination.^[^ ^18^ ^]^ Based on the above research considerations, we constructed an S‐scheme photocatalyst by first preparing an oxygen vacancy (V_O_)‐enriched ZnO substrate via calcination, followed by in situ hydrothermal growth of narrow‐bandgap Zn_0.5_Cd_0.5_S with robust reducing properties (ZnO‐V_O_@ZCS). The experimental findings demonstrate that V_O_ play a dual role as both electron reservoirs and transfer pathways, significantly accelerating charge transfer kinetics within the S‐scheme heterojunction. The synergy effects of the V_O_ and the S‐scheme charge transfer mechanism substantially enhance carrier separation and transmission efficiency within the heterojunction, while fully preserving the high oxidative potential of ZnO‐V_O_ and the strong reductive potential of ZCS. In addition, electron paramagnetic resonance (EPR) spectroscopy further verified that more V_O_ were generated at the heterojunction interface during its assembly process. The abundant V_O_ improved the material's photonic absorption capacity and surface adsorption site density, leading to a remarkable enhancement of the heterojunction's photocatalytic performance. Under acidic conditions (pH 4), the optimal ZnO‐V_O_@ZCS_0.20_ photocatalyst exhibited exceptional U(VI) removal efficiency, achieving 99.10% elimination within 10 min and a maximum U enrichment capacity of 1024.30 mg g^−1^ in 15 min. Furthermore, ZnO‐V_O_@ZCS_0.20_ was proven to have excellent U(VI) removal kinetics for diverse complex scenarios, being operational at wide pH ranges, at real sunlight irradiation, multi‐ionic/organic interference conditions, and for multi‐source aqueous systems (simulated nuclear wastewater, tap water, river water, and real U mine effluents). We believe that this work provides valuable insights for designing efficient S‐scheme heterojunctions and advancing the remediation of nuclear wastewater systems.

## 2 Results and Discussion

### 2.1 Material Structure and Composition Characterization

**Figure 1:**
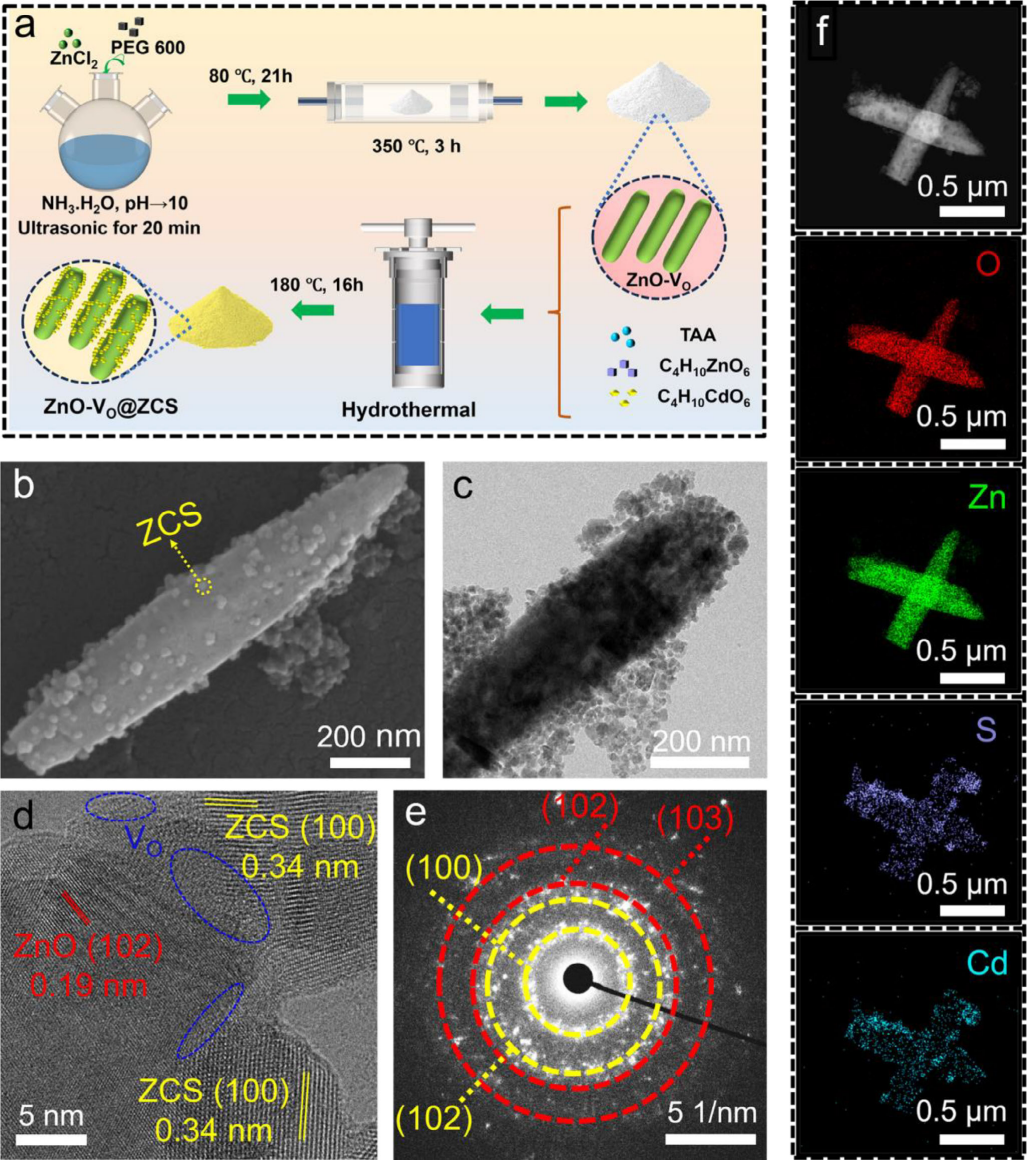
a) The schematic synthesis of ZnO‐V_O_@ZCS. b) SEM and c) TEM, d) HRTEM, and e) the SAED pattern images of ZnO‐V_O_@ZCS_0.20_, respectively. f) HAADF image and EDX elemental mappings of ZnO‐V_O_@ZCS_0.20_.

**Figure** **1**a shows the construction of the heterostructure of ZnO‐V_O_@ZCS. ZnO was first prepared using a solvothermal method, and then ZnO with V_O_ (ZnO‐V_O_) was obtained by annealing at 350 °C for 3 h. Finally, the ZnO‐V_O_@ZCS heterostructure was prepared by a simple and easily controlled in situ hydrothermal method (The details of the synthesis can be found in the Supporting Information). The morphology and microstructure of the synthesized samples were analyzed using scanning electron microscopy (SEM) and transmission electron microscopy (TEM). As illustrated in Figure S1a,b (Supporting Information), the ZnO‐V_O_ formed by the solvent method shows rod‐like structures, and ZCS alone consists of agglomerated nanoparticles. SEM and TEM images of ZnO‐V_O_@ZCS_0.20_ composites (Figure 1b,c) show that ZnO‐V_O_ is encapsulated by ZCS nanoparticles to form a tight heterojunction, indicating that the hydrothermal reaction does not change the morphology of ZnO‐V_O_, and that the formation of the junction can effectively prevent the aggregation of ZCS. Furthermore, high‐resolution TEM (HRTEM) images demonstrate a good combination of ZnO‐V_O_ and ZCS. It is observed that the lattice spacings of the (102) face of ZnO‐V_O_ and the (100) face of ZCS are 0.19 and 0.34 nm, respectively, and that a contact interface has been formed between ZnO‐V_O_ and ZCS (Figure 1d). In addition, significant lattice defects can be observed at the ZnO‐V_O_ surface and the heterojunction interface, indicating the presence of V_O_ (blue dashed circles). Multiple polycrystalline rings can also be observed in the selected area electron diffraction (SAED) model, which can be directed to the (102) and (103) faces of ZnO‐V_O_ and the (100) and (102) faces of ZCS, respectively, which provides additional evidence supporting its polycrystalline structure (Figure 1e). The high‐angle annular dark field (HAADF) and associated energy‐dispersive X‐ray spectroscopy (EDX) mapping results of ZnO‐V_O_@ZCS_0.20_ reveal the elemental composition and spatial distribution (Figure 1f).

The X‐ray diffraction (XRD) patterns of ZnO‐V_O_, ZCS, and ZnO‐V_O_@ZCS heterojunctions are shown in Figure S2 (Supporting Information). For the ZnO‐V_O_ and ZCS samples, it is found that all the diffraction peaks match well with the characteristic peaks of ZnO (PDF#89‐1397)^[^ ^19^ ^]^ and ZCS (PDF#89‐2943),^[^ ^20^ ^],^ respectively. Furthermore, as the ZCS content increased in the ZnO‐V_O_@ZCS composites, the characteristic diffraction peaks of the ZCS phase showed a progressive increase in intensity, and no extraneous peaks were detected in the XRD patterns. These observations conclusively demonstrate the successful synthesis of ZnO‐V_O_@ZCS composites without introducing any other impurities.

X‐ray photoelectron spectroscopy (XPS) was employed to investigate the elemental composition of the ZnO‐V_O_, ZCS, and ZnO‐V_O_@ZCS samples and the electron transfer process in the heterojunction. The XPS survey spectrum of ZnO‐V_O_@ZCS_0.20_ shows the coexistence of Zn, O, Cd, and S signals (Figure S3, Supporting Information). Upon further analysis, the high‐resolution XPS spectra revealed shifts in binding energies. As shown in Figure S4a (Supporting Information), the two XPS peaks near 1044.0 and 1021.0 eV can be assigned to Zn 2p_1/2_ and Zn 2p_3/2_, respectively. The Zn 2p binding energies of ZnO‐V_O_@ZCS_0.20_ were negatively and positively shifted compared to those of ZnO‐V_O_ and ZCS, respectively, indicating the close interfacial interactions between ZnO‐V_O_ and ZCS.^[^ ^21^ ^]^ The O1s spectrum of ZnO‐V_O_@ZCS_0.20_ in Figure S4b (Supporting Information) can be deconvoluted into three peaks: the peak at 529.98 eV is assigned to the Zn─O bond, the peak at 531.13 eV is related to the V_O_, and the peak at 532.20 eV is associated with the O─H bond in adsorbed water. ^[^ ^22^ ^]^ Additionally, the V_O_ of ZnO‐V_O_@ZCS_0.20_ exhibited higher relative peak intensities than those of ZnO‐V_O_, indicating higher V_O_ content in the complex. These may be because during ZCS formation, the S element displaces a small portion of O in ZnO‐V_O_, and the geometrical strain caused by the different atomic diameters of S and O leads to defects and lattice distortions—a phenomenon that also has been reported elsewhere.^[^ ^23^ ^]^ Meanwhile, the O 1s peak of the ZnO‐V_O_@ZCS_0.20_ sample is negatively shifted compared to the ZnO‐V_O_. The S 2p XPS profiles of the composite samples show that S 2p_3/2_ and 2p_1/2_ at 161.61 and 162.82 eV are shifted toward higher binding energies, respectively, as compared to the ZCS (Figure S4c, Supporting Information). For the high‐resolution Cd 3d spectrum of ZnO‐V_O_@ZCS_0.20_, two peaks with binding energies at 404.97 eV (Cd 3d_5/2_) and 411.72 eV (Cd 3d_3/2_), show the same peak shift trend as the hybrid S 2p XPS (Figure S4d, Supporting Information). From the above findings, it can be inferred that ZnO‐V_O_ and ZCS act as electron acceptor and donor in ZnO‐V_O_@ZCS, respectively, which implies that the charge transfer between ZnO‐V_O_@ZCS is likely to follow the S‐scheme pathway.^[^ ^24^ ^]^ It means that when *E*_f_ is balanced between these two semiconductors, the ZCS surface is positively charged, while ZnO‐V_O_ is the opposite, which results in an IEF directed from ZCS to ZnO‐V_O_. Under irradiation, the IEF directs the compounding of photogenerated carriers that are weakly redox‐competent, which accelerates the separation of the carriers and retains the optimal redox competence.

**Figure 2:**
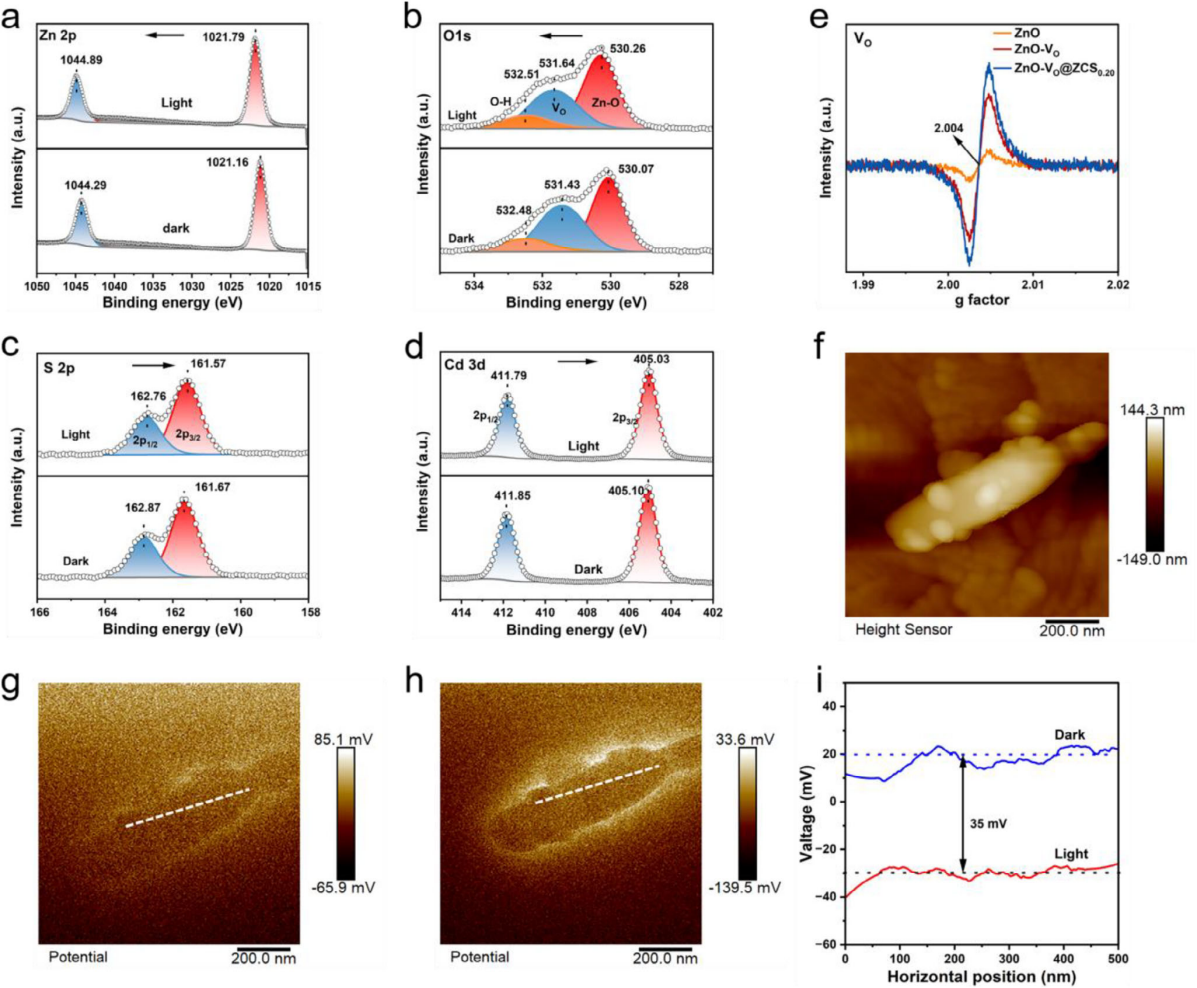
In situ XPS spectra before and after light irradiation of a) Zn 2p, b) O 1s, c) S 2p, and d) Cd 3d for the ZnO‐V_O_@ZCS_0.20_ heterojunction photocatalyst. e) EPR spectra of unannealed ZnO, annealed ZnO‐V_O_, and ZnO‐V_O_@ZCS_0.20_ synthesized via in situ hydrothermal method. f) AFM topographic image of ZnO‐V_O_@ZCS_0.20_. g) KPFM images of ZnO‐V_O_@ZCS_0.20_ in darkness. h) KPFM image of ZnO‐V_O_@ZCS_0.20_ with 365 nm UV light illumination. i) The corresponding surface potential profiles of ZnO‐V_O_@ZCS_0.20_ in the dark and with 365 nm UV light illumination.

The charge transfer kinetics of ZnO‐V_O_@ZCS_0.20_ under light irradiation were studied by in situ XPS analysis, which verified the above speculations. As shown in **Figure** **2**a–d, distinct binding energy shifts are observed where the O 1s and Zn 2p core levels show upward shifts toward higher binding energies, whereas the S 2p and Cd 3d orbitals display downward shifts toward lower binding energies. This indicates that photoelectrons are transferred from ZnO‐V_O_ to ZCS when both of them are excited at the same time, which is consistent with the S‐scheme charge transfer pattern. The presence of V_O_ in ZnO‐V_O_ accelerates this transfer process because V_O_ can capture electrons and thus improve charge separation efficiency.^[^ ^16^ ^]^ The EPR spectrum exhibits a signal at *g* = 2.004, corresponding to V_O_ in the sample. The unannealed ZnO exhibits a negligible signal, significantly weaker than those of annealed ZnO‐V_O_ and ZnO‐V_O_@ZCS_0.20_. Notably, the V_O_ signal for the composite ZnO‐V_O_@ZCS_0.20_ is slightly stronger than that of ZnO‐V_O_, indicating that the annealing process and hydrothermal reaction increase the V_O_ concentration (Figure 2e).

To further demonstrate that the photogenerated charge at the ZnO‐V_O_@ZCS_0.20_ heterojunction interface follows the S‐scheme electron transfer mechanism, light‐assisted Kelvin probe force microscopy (KPFM) was employed to probe surface potential changes of the heterojunction under dark and illuminated conditions. As shown in Figure 2f, the AFM images of ZnO‐V_O_@ZCS_0.20_ reveal ZCS particles uniformly distributed on ZnO‐V_O_. Figure 2g,h present KPFM images of ZnO‐V_O_@ZCS_0.20_ under dark and 365 nm UV illumination, respectively. The corresponding quantitative measurements of the surface potential are shown in Figure 2i. Under light conditions, the ZnO‐V_O_@ZCS_0.20_ surface potential decreases compared to dark conditions, with the surface potential of the sample being ≈ 20 mV in the absence of light, whereas the surface potential at the same locations in the sample decreases to ≈−30 mV under irradiation. This negative shift in surface potential underillumination indicates the accumulation of photo‐generated electrons at the surface. ^[^ ^25^ ^]^ Given that ZCS covers the photocatalyst's outer surface, this observation suggests that ZCS functions as an electron acceptor during illumination. These results align with in situ XPS data, collectively confirming an S‐scheme charge transfer mechanism.

### 2.2 Optical Properties and Charge Transfer Analysis

**Figure 3:**
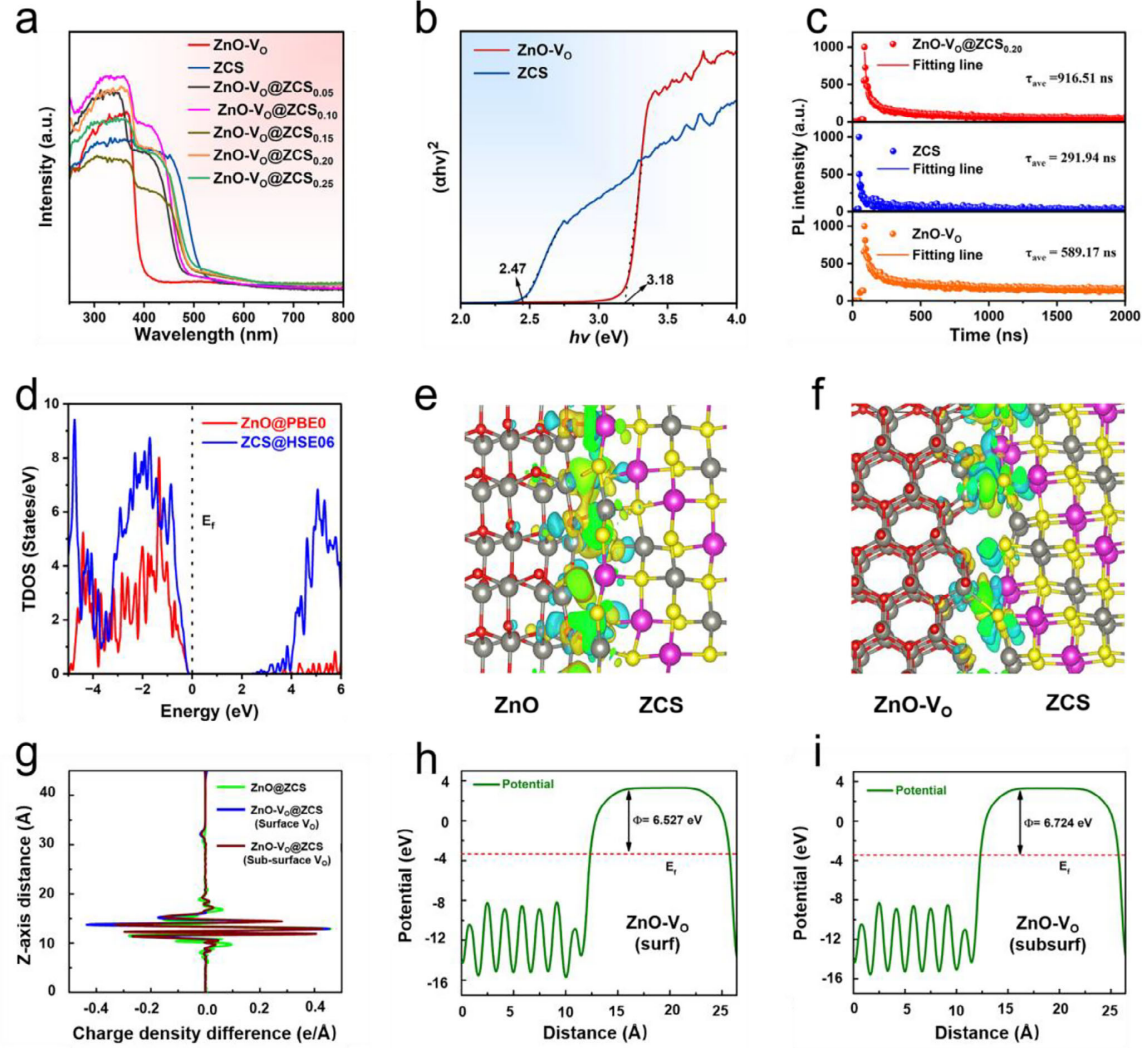
a) UV–vis DRS spectra of ZnO‐V_O_, ZnS, and ZnO‐V_O_@ZCS composites. b) Tauc plots of ZnO‐V_O_ and ZCS. c)Time‐resolved photoluminescence decay profiles of ZnO‐V_O_, ZCS, and ZnO‐V_O_@ZCS_0.20_. Theoretical calculations: d) Calculated TDOS for bulk ZnO and ZnCdS. (e) 3D isosurface for calculated charge density difference at ZnO@ZCS interface of 1 × 1 unit cell, where charge density isosurface is set to 0.002 e Å^3^. f) 3D isosurface for calculated charge density difference at ZnO‐V_O_@ZCS interface of 2  ×  1 unit cell (with ZnO surface V_O_), where charge density isosurface is set to 0.002 e Å^3^. g) 1D planar average charge density difference across the interface for ZnO@ZCS and ZnO‐V_O_@ZCS. Calculated potential energy and work function for h) (110) 2  ×  2 supercell slab with surface‐V_O_ in ZnO at the PBE0 level of theory, and i) (110) 2  ×  2 supercell slab with sub‐surface‐V_O_ in ZnO at the PBE0 level of theory.

Ultraviolet‐visible diffuse reflectance spectra (UV–vis DRS) revealed the light absorption capacity of the prepared samples. As shown in **Figure** **3**a, the absorption edge of pure ZnO‐V_O_ is at 410 nm, and that of ZCS is at 525 nm. After loading ZCS, the light absorption of composite ZnO‐V_O_ is red‐shifted. Noteworthy, all ZnO‐V_O_@ZCS composites showed enhanced absorption in the 500–800 nm range compared to pure ZnO‐V_O_ and ZCS, which may be attributed to the presence of more V_O_ in ZnO‐V_O_@ZCS.^[^ ^26^ ^]^ Moreover, the corresponding Tauc plot analysis reveals bandgap energies (*E*_g_) of 3.18 eV for ZnO‐V_O_ and 2.47 eV for ZCS, respectively (Figure 3b).^[^ ^27^ ^]^ The flat‐band potentials (*E*_fb_) of ZnO‐V_O_ and ZCS (versus Ag/AgCl) are determined via Mott–Schottky (M‐S) analysis to be −0.75 and −0.97 V, respectively (Figure S5, Supporting Information). These values were converted to the standard hydrogen electrode (NHE) scale using the equation *E*_fb_ (versus NHE) = *E*_fb_ (Ag/AgCl) + 0.197 V, yielding *E*_fb_ values of −0.55 V and −0.77 V for ZnO‐V_O_ and ZCS (versus NHE), respectively. Furthermore, the observed positive slope tendency in the M‐S plots confirms the n‐type semiconducting characteristics of both materials.^[^ ^28^ ^]^ Generally, the value of *E*_fb_ is ≈0.2 eV higher than the conduction band (CB) potential for an n‐type semiconductor.^[^ ^29^ ^]^ Thus, it can be inferred that the CB of ZnO‐V_O_ and ZCS are −0.75 and −0.97 eV (vs NHE), respectively. Based on the formula for the valence band (VB) potential: *E*_VB_ = *E*_g_ − *E*_CB_, the *E*_VB_ of ZnO‐V_O_ and ZCS can be calculated as 2.43 and 1.50 eV, respectively. Meanwhile, the VB‐XPS values (*E*_VB, XPS_) of ZnO‐V_O_ and ZCS were measured as 2.66 and 1.70 eV, respectively (Figure S6, Supporting Information). Then, the *E*_VB, XPS_ were converted to the VB values (*E*_VB, NHE_) at the standard hydrogen electrode according to the equation *E*_VB, NHE_ = φ + *E*_VB, XPS_ ‐ 4.44, where φ is the instrumental work function (4.2 eV). The VB values of 2.43 and 1.46 eV for ZnO‐V_O_ and ZCS, respectively, demonstrate good agreement with both the M‐S analysis results and the bandgap extrapolation data.

The charge transfer dynamics of ZnO‐V_O_, ZCS, and ZnO‐V_O_@ZCS were systematically characterized through steady‐state photoluminescence (PL) spectroscopy, time‐resolved photoluminescence (TRPL) decay profiles, electrochemical impedance spectroscopy (EIS), and transient photocurrent responses. As shown in Figure S7 (Supporting Information), the PL spectral intensity of ZnO‐V_O_@ZCS_0.20_ is significantly reduced compared to ZnO‐V_O_ and ZCS, confirming enhanced photogenerated carrier separation.^[^ ^30^ ^]^ TRPL analysis further showed prolonged carrier lifetimes in ZnO‐V_O_@ZCS_0.20_ (Figure 3c; Table S1, Supporting Information), suggesting a slowdown of electron and hole complexation after the heterojunction formation.^[^ ^31^ ^]^ Furthermore, the EIS spectrum showed that the Nyquist arc radius of ZnO‐V_O_@ZCS_0.20_ was significantly reduced (Figure S8, Supporting Information), which indicates that the composite has a low charge transfer resistance and better conductive ability.^[^ ^32^ ^]^ As further evidence, the transient photocurrent response recorded from multiple photo‐switching cycles clearly shows that the photocurrent density of ZnO‐V_O_ and ZCS composite is greater than that of the individual ones (Figure S9, Supporting Information), which further proves that the ZnO‐V_O_@ZCS_0.20_ has a higher charge transfer efficiency.

To understand the experimental results, we first analyze the electronic properties of ZnO and ZCS. Densities of states (DOS) of bulk ZnO and ZCS calculated with the hybrid PBE0 and HSE06 functionals, respectively, are shown in Figure 3d. It was revealed that the range‐separated HSE06 hybrid functional underestimated the bandgap energy value of ZnO (2.47 eV), whereas the hybrid PBE0 functional resulted in a reasonable bandgap of 3.16 eV, which correlates well with the experimental data. The predicted underestimated HSE06 bandgap value is also in line with previously reported results.^[^ ^33^ ^]^ In the case of ZCS, the unscreened hybrid PBE0 functional resulted in an overestimated bandgap of 2.99 eV; however, the HSE06 predicted bandgap of 2.37 eV is found to agree with the measured experimental data (2.47 eV).

Experimentally, it was proven that ZnO contains V_O_. Such vacancies are likely to affect catalytic properties due to ZnO electronic structure tuning that could result in improved charge dynamics at the interface. When ZnO forms an interface with other materials, V_O_ predominantly emerge as surface and subsurface defects rather than bulk defects. This preference is primarily driven by differences in thermodynamic stability, chemical interactions, and vacancy formation energy. To get insight into the surface V_O_ in ZnO‐V_O_, the formation energy (*E^f^*) is calculated by removing an oxygen atom from the surface layer (Figure S10, Supporting Information). The formation energy for neutral V_O_ is dependent on the chemical potential of oxygen as well as on the total energy of the perfect structure and the V_O_‐based structure. In case of a charged vacancy, it again depends on the Fermi energy level. The well‐known expression for *E^f^* calculations is given somewhere else.^[^ ^34^, ^35^, ^36^ ^]^ Using DFT, the *E^f^* of 2 × 2 supercell (110) surface of ZnO‐V_O_ is calculated to be 2.75/3.06 eV at the PBE/PBE0 levels. These results match well with the previous ones (2.72 eV) reported by Cantele et al.^[^ ^37^ ^]^ For deeper vacancy positions, i.e., at the sub‐surface level (Figure S10, Supporting Information), the formation energies are 3.46/3.88 eV at the PBE/PBE0 theory level, respectively. These values also demonstrate good agreement with the reported *E^f^* of 3.37 eV calculated at the PBE level.^[^ ^37^ ^]^ For bulk ZnO‐V_O_, the reported *E^f^* is 3.9 eV.^[^ ^34^ ^]^ These results explain that V_O_ are prone to form on the surface and subsurface levels more easily compared to the bulk due to the under‐coordination of atoms, which makes oxygen loss more favorable. These regions experience significant interactions with the interfacing material, such as charge transfer and chemical bonding, which can further lower the formation energy of vacancies. Consequently, V_O_ accumulate at or near the surface, especially under reducing conditions or during high‐temperature processing. Using self‐consistent potential correction for charged periodic systems,^[^ ^38^ ^]^ we found that +1 charged O vacancies are more prone to form at the ZnO surface and subsurface levels (*E^f^* are ≈0.25 eV lower compared to neutral ones). However, the formation of the interface induces drastical change making neutral vacancies energetically more favorable compared to +1 and +2 charged vacancies with *E^f^* equal to 3.66 and 3.75 eV (at the PBE level) for surface and subsurface O vacancies, respectively. We believe that this effect could be associated with charge redistribution upon the heterostructure formation. The results confirm the idea that the oxygen vacancies can accept electrons and enhance the charge separation characteristics.

In order to analyze the charge transfer behavior at the interface of the heterostructure, we calculated the charge density for the heterostructure and individual semiconductors, as well as performing a Bader charge analysis. The charge density calculation for the heterostructure is performed with the PBE0 method. A 3D isosurface for charge density difference at the interface for the heterostructure slab is presented in Figure 3e,f. This charge density difference is quantitatively calculated by the following formula.

(1) $$\Delta \rho =\rho_{ZnO@ZCS}-\rho_{ZnO}-\rho_{ZCS}$$

Here, charge accumulation and depletion are shown by yellow and cyan colors, respectively. We observe that a significant amount of charge transfer occurs through Zn‐S interaction, whereas comparatively less occurs through the Zn─O and Cd─O interactions at the heterojunction. The calculated 1D planar average charge density difference at the interface (Figure 3g) provides a quantitative understanding of the amount of charge transfer and direction of charge transfer at the heterojunction. Additionally, this explains that the charge density variation is principally localized near the interface (the calculated positive and negative values represent electron accumulation and depletion, respectively). From the Bader charge analysis, we confirm that 0.32|*e*| transferred across the non‐defective interface. In case of ZnO‐V_O_@ZCS, this charge transfer is found to be increased by 0.08|*e*| and 0.05|*e*| with V_O_ at ZnO surface and sub‐surface, respectively. Moreover, it is to be noted that the ZnO surface V_O_ promoted more charge transfer (i.e., 0.40|*e*|) than the sub‐surface V_O_ (0.37|*e*|). So, it is clear that V_O_ in ZnO increased the interfacial charge transfer and promoted the S‐scheme behavior of the ZnO‐V_O_@ZCS heterostructure.

The work function (Φ) calculations in Figure S11a,b (Supporting Information) and Figure 3h,i explain the charge transfer characteristics of the materials. It can be seen that ZnO possesses a higher work function value, i.e., 7.195 eV than that of ZCS (6.372 eV), and that the Fermi energy level of the former is lower than the latter. This difference in work functions suggests that when two semiconductors are in contact, an internal electric field is created and electrons flow from ZCS to ZnO material. This electric field facilitates the formation of an S‐scheme charge transfer mechanism. Additionally, the work function (Φ) is very sensitive to the point defects on the surface. To focus on this, we calculated Φ for the 2 × 2 supercell (110) surface slab ZnO‐V_O_ structure for V_O_ at the surface and sub‐surface levels. We found that the creation of V_O_ induced a negative change in the work function, which implies a 0.66 eV lower work function value, i.e., Φ = 6.527 eV, than that of ZnO surface (*Φ* = 7.187 eV, for 2 × 2 supercell). For V_O_ at the sub‐surface level, Φ is calculated to be 6.724 eV. So, it can be noted that though the presence of V_O_ reduces the work function of ZnO, it is still higher than that of ZCS, and that the difference facilitates the S‐scheme charge transfer. The higher CB edge of ZCS (−0.97 V versus NHE) makes it an efficient reducing photocatalyst, while the deeper VB edge of ZnO (2.45 V versus NHE) reflects its strong oxidation capability. Upon light excitation, both ZnO and ZCS generate photoexcited electrons that transit to their respective CBs. The built‐in electric field drives electrons from the ZnO's CB to recombine with holes in the ZCS's VB. This S‐scheme heterojunction charge transfer mechanism not only improves the separation of the photoexcited carriers compared to type‐II transfer but also enhances the redox capability.

**Figure 4:**
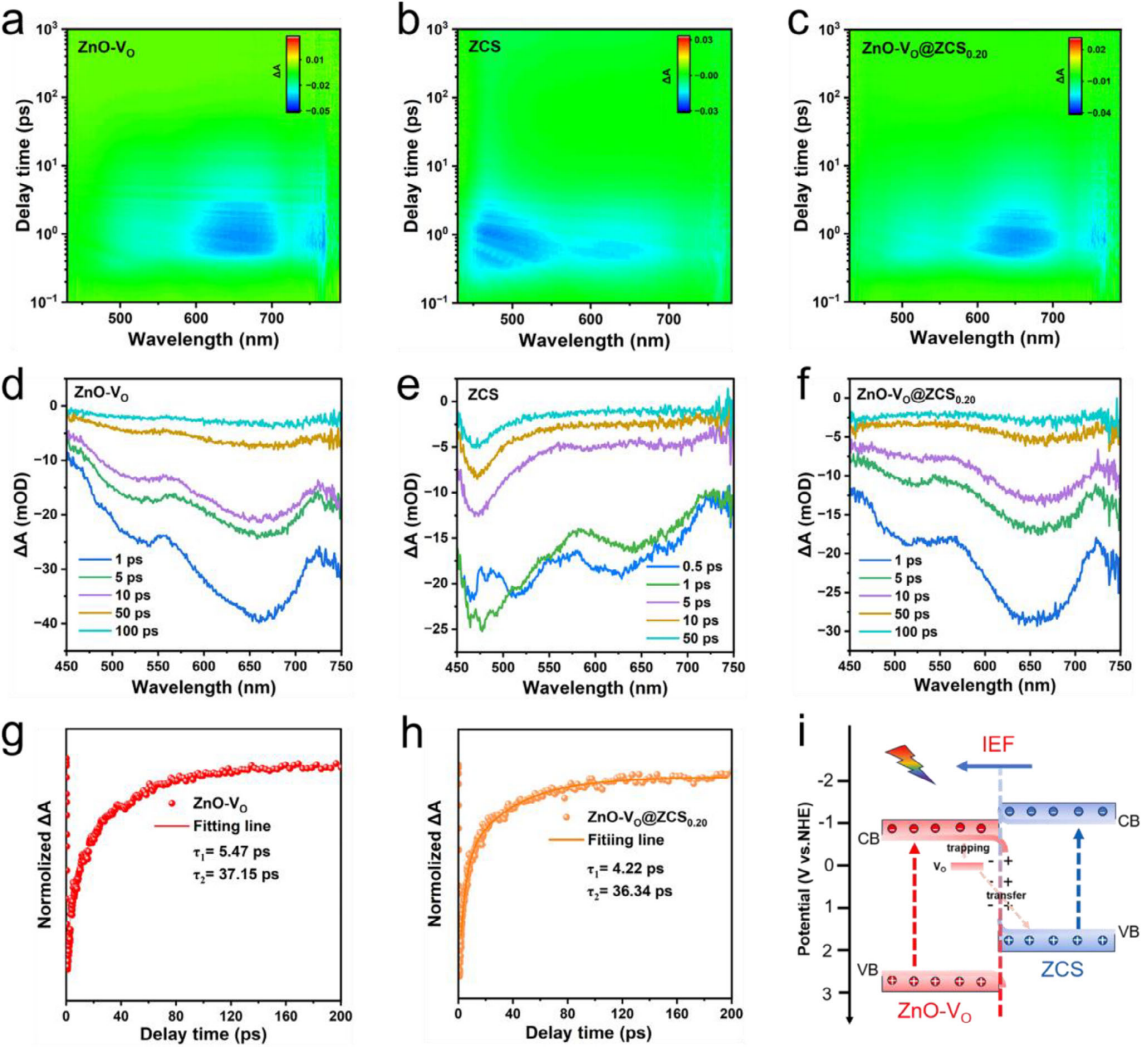
The 2D fs‐TA spectra of a) ZnO‐V_O_, b) ZCS, c) ZnO‐V_O_@ZCS_0.20_. The fs‐TA spectra at different decay times of d) ZnO‐V_O_, e) ZCS, and f) ZnO‐V_O_@ZCS_0.20_. The Normalized decay kinetic curves of g) ZnO‐V_O_, (h) ZnO‐V_O_@ZCS_0.20_, and i) the proposed photoinduced electron transfer route of ZnO‐V_O_@ZCS_0.20_ heterojunction.

Furthermore, fs‐TAS was employed to investigate the regulatory mechanisms of V_O_ on carrier dynamics in the ZnO‐V_O_@ZCS S‐scheme heterojunction under photoexcitation. **Figure** **4**a–c presents the 2D pseudo‐color maps of ZnO‐V_O_, ZCS, and ZnO‐V_O_@ZCS_0.20_. The corresponding spectra at different delay times are shown in Figure 4d–f. Clearly, ZnO‐V_O_, ZCS, and ZnO‐V_O_@ZCS_0.20_ samples all exhibit broad negative absorption signals in the visible region, assigned to the ground‐state bleaching (GSB) of photoelectrons in their CB, where the signal of ZnO‐V_O_@ZCS_0.20_ comes from the joint contribution of the signals of ZnO‐V_O_ and ZCS. The decay curves of ZnO‐V_O_ and ZnO‐V_O_@ZCS_0.20_ are fitted separately using the double‐exponential equation [(*Δ*A = A_1_ exp(‐t/τ_1_) + A_2_ exp(‐t/τ_2_)], where the short time τ_1_ and long time τ_2_ correspond to the photogenerated electrons captured by the shallow and deep capture states, respectively. As shown in Figure 4g,h, ZnO‐V_O_@ZCS_0.20_ exhibits reduced lifetimes (*τ*_1_ = 4.22 ps, *τ*_2_ = 36.34 ps) compared to ZnO‐V_O_ (*τ*_1_ = 5.47 ps, *τ*_2_ = 37.15 ps), suggesting that an additional channel for electron transfer exists at the interface between ZnO‐V_O_ and ZCS, accelerating the relaxation process of electrons. Based on this analysis, we propose th mechanism of V_O_ action in the ZnO‐V_O_@ZCS S‐scheme heterojunction as shown in Figure 4i. After irradiation, the shallowly trapped and active photoelectrons are stored in the defect levels and then rapidly induce electron transfer to ZCS. The swift electron trapping, as well as the fast charge separation at the interface of ZnO‐V_O_@ZCS, suppresses electron‐hole recombination and greatly enhances the efficiency of charge separation. ^[^ ^16^ ^]^

### 2.3 Photocatalytic U(VI) Removal

**Figure 5:**
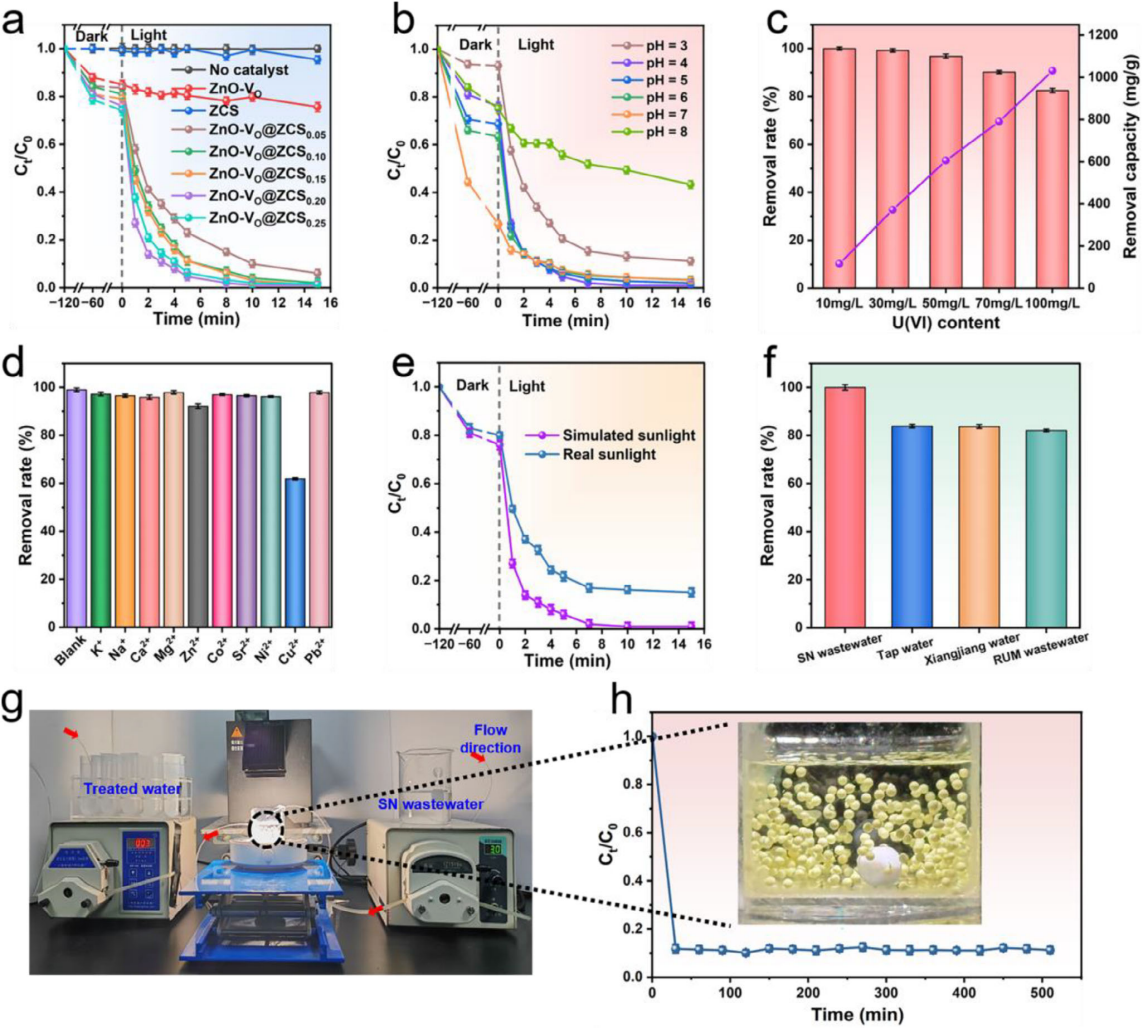
a) Photocatalytic U(VI) removal over different catalysts (experiment conditions: m/V = 0.08 g L^−1^, C_U(VI)_ = 30 mg L^−1^, pH 4, T = 298 K). Effect of varying b) pH conditions, c) U(VI) initial concentrations, and d) co‐existing metal ions on ZnO‐V_O_@ZCS_0.20_ for U(VI) removal. e) The performance of ZnO‐V_O_@ZCS_0.20_ for U(VI) removal under different light sources. f) The performance of ZnO‐V_O_@ZCS_0.20_ for U(VI) removal in the U‐spiked simulated nuclear (SN) wastewater, tap water, Xiangjiang water and real U mine (RUM) wastewater (SN wastewater: C _U(VI)_ = 28.8 mg L^−1^, C _Ni(II)_ = 22 mg L^−1^, C_Cu(II)_ = 12 mg L^−1^, C_Ca(II)_ = 3 mg L^−1^, C_Mg(II)_ = 0.1 mg L^−1^, C_Pb(II)_ = 0.1 mg L^−1^). g) The continuous‐flow photocatalytic reactor unit. h) The Change of effluent U (VI) concentration per 30 min.

The removal performance of the prepared sample for U(VI) was further investigated. **Figure** **5**a shows reaction time curves of ZnO‐V_O_, ZCS, and ZnO‐V_O_@ZCS to U(VI) under simulated sunlight irradiation and non‐irradiation conditions. Before turning on the light, the solution was stirred in a dark environment for 2 h to bring the reaction system to adsorption‐desorption equilibrium for U(VI). The extraction of U(VI) by ZnO‐V_O_@ZCS in the dark was enhanced as compared to that of pristine ZnO‐V_O_ and ZCS, which can be explained by the abundant V_O_ within the heterojunction structure that provide increased adsorption sites for U(VI) immobilization. Specifically, ZnO‐V_O_@ZCS samples can remove ≈20% of U(VI) after 2 h of dark adsorption, indicating a slow adsorption process. The enrichment of U(VI) facilitates the photocatalytic reduction and realizes the synergistic effect of adsorption and photoreduction.^[^ ^39^, ^40^ ^]^ After the introduction of the light source, the removal rate of U(VI) by ZnO‐V_O_@ZCS was greatly increased, in fact, the best catalytic performance of ZnO‐V_O_@ZCS_0.20_ could remove 99.10% of U(VI) within 10 min, which was 4.0 and 20.0‐fold higher than that of ZnO‐V_O_ (24.38%) and ZCS (4.95%), respectively. This confirms that U(VI) elimination occurs primarily through photocatalysis, and that its performance surpasses the most recently reported photocatalysts (Table S2, Supporting Information). In addition, the photocatalytic rates of all the ZnO‐V_O_@ZCS composites are higher than those of ZnO‐V_O_ and ZCS, and the maximum catalytic reaction rate constant *k* was 0.4114 min^−1^, which is far greater than that of ZnO‐V_O_ (0.0055 min^−1^) and ZCS (0.00009 min^−1^), respectively (Figure S12, Supporting Information). To verify the crucial role of V_O_ in the catalytic reaction, we compared the photocatalytic performance of the ZnO@ZCS_0.20_ and ZnO‐V_O_@ZCS_0.20_ composites. As shown in Figure S13 (Supporting Information), the ZnO‐V_O_@ZCS heterojunction exhibited significantly enhanced U(VI) photoreduction efficiency compared to the ZnO@ZCS composite with the same ZCS content. These findings demonstrate that the S‐scheme heterojunction composites with specific electron transfer channels can significantly improve the photocatalytic performance. Notably, ZnO‐V_O_@ZCS_0.20_ was selected to further evaluate its U(VI) removal ability due to its best performance among the prepared ZnO‐V_O_@ZCS samples.

The existence of species of U and the surface charge of the catalyst in solution will change with pH, leading to a change in the U‐extraction performance of the catalyst. Therefore, it is essential to explore the applicable pH range of the material. Figure 5b and Figure S14 (Supporting Information) show the removal performance of ZnO‐V_O_@ZCS_0.20_ for U(VI) removal in the pH range from 3 to 8. Surprisingly, ZnO‐V_O_@ZCS_0.20_ exhibits a high removal capacity (>90%) and high reaction rate under weakly acidic and neutral conditions (3 ≤ pH ≤ 7), which is similar to the pH range of nuclear wastewater.^[^ ^41^ ^]^ It could be explained by the fact that at low pH (pH ≤ 7), positively charged cations are the main form of U, such as UO_2_^2+^, UO_2_(OH)^+^, and (UO_2_)_2_(OH)_2_^2+^.^[^ ^42^ ^]^ The results of the surface zero‐point charge test of ZnO‐V_O_@ZCS_0.20_ show that the catalyst displays a negative charge at the experimental pH conditions and will be mutually attracted to the positively charged U species (Figure S15, Supporting Information), thus promoting adsorption and catalysis. However, its catalytic performance was weakened under an alkaline environment (pH 8). That's because, at pH > 7, U exists mainly in the form of anions, such as UO_2_(OH)_3_^−^ and (UO_2_)_3_(OH)_7_^−^.^[^ ^43^ ^]^ The electrostatic repulsion between the catalyst and the anion increases, and the adsorption slows down, leading to poor catalytic performance.

To avoid the waste of catalyst, the suitable solid‐liquid ratio was determined, as illustrated in Figure S16 (Supporting Information). With the increase of the dosage of ZnO‐V_O_@ZCS_0.20_, the removal rate of U(VI) gradually increases. When the solid‐liquid ratio reaches 0.08 g L^−1^, the removal rate of U can reach up to 99.10% with the maximum *k* value of 0.4114 min^−1^. Furthermore, the removal capacity of ZnO‐V_O_@ZCS_0.20_ for U was evaluated by increasing the concentration of U(VI) (Figure 5c). Specifically, the removal rate of ZnO‐V_O_@ZCS_0.20_ for U(VI) decreased with increasing concentration at an initial concentration ranging from 10 to 100 mg L^−1^, while the extraction of U gradually increased. Notably, ZnO‐V_O_@ZCS_0.20_ achieved up to 1024.30 mg g^−1^ of U extraction capacity within 15 min under simulated sunlight irradiation at an initial U(VI) concentration of 100 mg L^−1^.

Because nuclear wastewater has a complex composition, containing a variety of cations, anions, and organic pollutants, these components may compete with U(VI) for catalytically active sites, which has a great impact on U(VI) removal. Therefore, we further explored the interference resistance and stability of ZnO‐V_O_@ZCS_0.20_. First, we evaluated the U(VI) removal capability of ZnO‐V_O_@ZCS_0.20_ under the competitive interference of multiple metal cations and common anions. As depicted in Figure 5d and Figure S17 (Supporting Information), ZnO‐V_O_@ZCS_0.20_ maintained > 92% U(VI) removal rate under competition and interference from different metal cations (except for Cu^2+^) and anions. The effect of competition between Cu^2+^ and U(VI) for the active site may have contributed to its inhibition of the material's ability to capture U(VI), resulting in a removal rate of only 61.8%. However, the amount of Cu^2+^ in real nuclear wastewater is much lower than that of U(VI), so the sample still has the potential for effective practical application. In addition, we further evaluated the effectiveness of ZnO‐V_O_@ZCS_0.20_ in removing and reducing U(VI) under the co‐existence of different organic pollutants such as Rhodamine B (RHB), Crystal violet (CV), and Methylene blue (MB) (Figure S18, Supporting Information). It can be seen that when the catalyst co‐exists with organic pollutants, the removal rate of U(VI) decreases slightly, but the removal rate can still reach more than 85%, and the organic pollutants can be removed at the same time. The decrease in the removal rate of U(VI) may be caused by the competition between organic matter and U(VI) for catalytically active sites and free radicals. Moreover, direct solar‐driven photocatalysis has been a sought‐after potential application area from the point of view of economic benefits. To this end, the photoreduction of U(VI) was tested under natural light (Hengyang, China, November 7th, 2024, 11:00 am). As expected, the photocatalytic reaction performed well under solar energy, and 86.7% of U(VI) was rapidly removed within 15 min, demonstrating its excellent solar‐driven catalytic performance (Figure 5e).

To further demonstrate the potential application of ZnO‐V_O_@ZCS_0.20_ in treating real nuclear wastewater. U‐spiked simulated nuclear (SN) wastewater, tap water, Xiangjiang River water, and real U mine (RUM) wastewater (from pore water of tailings in a mine located in Hunan Province, China; its main components and parameters are shown in Table S3, Supporting Information) were configured for experiments (Figure 5f). Noteworthy, ZnO‐V_O_@ZCS_0.20_ was put into the formulated SN wastewater containing multiple ion species for photocatalytic experiments and also showed good U(VI) removal behavior with a removal rate over 99.0%. However, the removal of U(VI) in the U‐spiked tap water, Xiangjiang River water, and RUM wastewater only reached 83.93%, 83.75%, and 82.10%, respectively, which might be caused by the presence of plankton and different kinds of complex ions in these two water samples. These findings verified that ZnO‐V_O_@ZCS_0.20_ is an ideal catalyst candidate for the efficient removal of U(VI) in complex U wastewater environments. To address catalyst recovery as well as to evaluate the catalytic potential of ZnO‐V_O_@ZCS_0.20_ in SN wastewater, ZnO‐V_O_@ZCS_0.20_/SA gel spheres were fabricated by incorporating the photocatalyst into a sodium alginate (SA) matrix, accompanied by a simple continuous‐flow photocatalytic reactor for the experimental validation. As shown in Figure 5g,h, throughout the experimental setup, the water flow was controlled at 0.75 mL min^−1^ by a flow pump, and 0.5 L of configured simulated uranium wastewater was continuously pumped into a reactor containing recoverable ZnO‐V_O_@ZCS_0.20_/SA gel spheres. An Xe lamp was used here as a light source to simulate the sunlight for the continuous recoverable uranium extraction experiments. Sampling and testing of U(VI) residual concentrations were conducted at half‐hourly intervals. The results show that U(VI) can be continuously reduced in this process, and that the removal rate remained ≈90%, indicating that ZnO‐V_O_@ZCS_0.20_ has high stability and potential for practical application.

### 2.4 Photocatalytic Product and Reaction Mechanism Analysis

**Figure 6:**
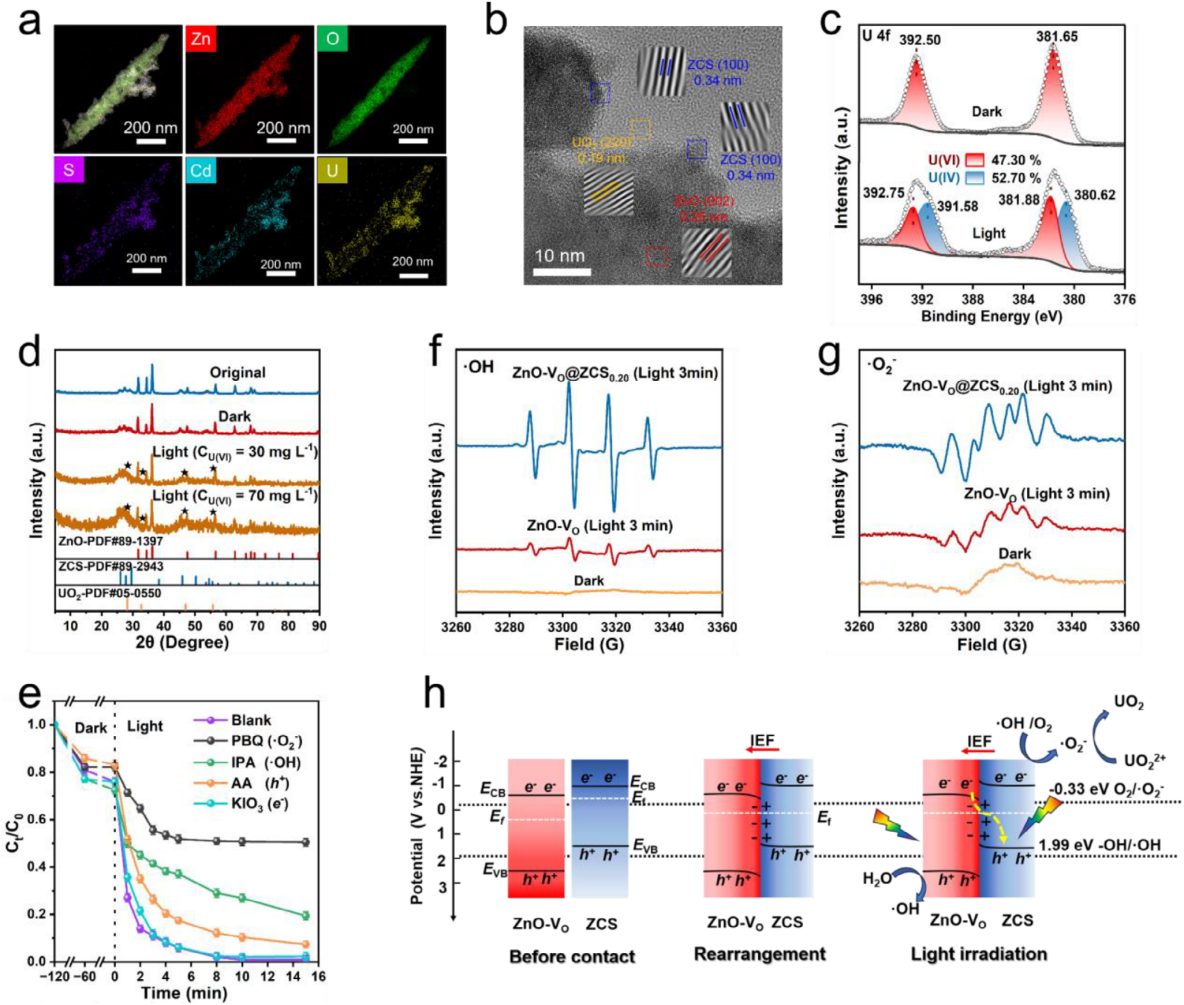
a) TEM image and the corresponding elemental mappings, b) HRTEM micrograph of ZnO‐V_O_@ZCS_0.20_ after the catalytic reaction. c) High‐resolution XPS spectra of U4f of ZnO‐V_O_@ZCS_0.20_ under dark and light conditions. d) XRD patterns of ZnO‐V_O_@ZCS_0.20_ at different stages in the photocatalytic reaction. e) Effects of various scavengers on the U(VI) removal efficiency over ZnO‐V_O_@ZCS_0.20_. EPR signals of f) DMPO‐⋅OH, and g) DMPO‐⋅O_2_^−^. h) Schematic illustration of charge carrier migration on ZnO‐V_O_@ZCS under the photocatalytic removal process of U(VI).

To elucidate the photocatalytic products and evaluate the stability of the catalyst, comprehensive structural and compositional characterizations of ZnO‐V_O_@ZCS_0.20_ were performed before and after the photocatalytic reaction. TEM elemental mapping analysis (**Figure** **6**a) combined with EDS measurements (Figure S19, Supporting Information) revealed that the catalyst maintained its structural stability throughout the photocatalytic process. The elemental mapping demonstrated a homogeneous distribution of U species on the catalyst surface, alongside the characteristic elements of the catalyst (O, Zn, Cd, and S), indicating successful U immobilization. HRTEM examination (Figure 6b) identified distinct lattice spacings of 0.26, 0.34, and 0.19 nm, corresponding to the (002) plane of ZnO‐V_O_, the (100) plane of ZCS, and the (220) plane of UO_2_, respectively. Furthermore, FTIR analysis (Figure S20, Supporting Information) revealed the emergence of a characteristic vibrational band at ≈900 cm^−1^, which is attributed to the O = U = O stretching mode, providing additional evidence for U species incorporation on the catalyst surface.^[^ ^44^ ^]^

The chemical states and structural evolution of the catalyst were further investigated through XPS and XRD analyses. The XPS survey spectrum (Figure S21, Supporting Information) and high‐resolution spectra (Figure S22, Supporting Information) confirmed the presence of characteristic peaks corresponding to Zn 2p, O 1s, S 2p, Cd 3d, and U 4f after the photocatalytic reaction. The observed binding energy shifts in these elements suggest potential modifications in their local chemical environments, likely induced by the absorption of U. Notably, after dark adsorption, the U 4f spectrum showed only U(VI) peaks (381.65 eV, 392.50 eV), whereas after photocatalysis, four distinct peaks appeared corresponding to U(VI) (381.88 eV, 392.75 eV) and U(IV) (380.62 eV, 391.58 eV). Quantitative analysis revealed that 52.70% of surface uranium exists as U(IV), confirming efficient photocatalytic reduction of U(VI) to U(IV) (Figure 6c).

XRD patterns (Figure 6d) indicated that the ZnO‐V_O_@ZCS_0.20_ catalyst maintained its structural integrity without detectable phase transformation after the photocatalytic reaction. Although the diffraction peaks of UO_2_ (PDF#05‐0550) overlapped with those of ZCS, comparative analysis of samples treated with different initial U(VI) concentrations (30 versus 70 mg L^−1^) revealed enhanced intensity of UO_2_ characteristic peaks at 28.24 °, 32.72 °, 46.94 °, and 55.70 °, corresponding to the (111), (200), (220), and (311) crystal planes, respectively. This concentration‐dependent intensity increase further corroborates the successful formation of UO_2_ on the catalyst surface.

The catalyst's practical applicability was further evaluated through cycling experiments, which demonstrated remarkable stability with over 80% U(VI) removal efficiency maintained after five consecutive cycles (Figure S23, Supporting Information). This decrease in catalytic performance may be due to photodegradation, but the reduction is still within an acceptable range.^[^ ^45^, ^46^ ^]^ This finding highlights the material's excellent chemical stability and recyclability, making it a promising candidate for practical U extraction applications.

Radical scavenger experiments were conducted to better understand the photocatalytic reduction mechanism of U(VI) over ZnO‐V_O_@ZCS_0.20_ under simulated irradiation. The photocatalytic system was treated with specific scavengers, including ascorbic acid (AA) for holes (*h* ^+^) scavenging, tert‐butanol (TBA) for ·OH scavenging, p‐benzoquinone (p‐BQ) for ·O_2_^−^ scavenging, and KIO_3_ for photoinduced electrons (*e* ^−^) scavenging, to identify the predominant reactive oxygen species involved in the photocatalytic process. As illustrated in Figure 6e, the addition of KBrO_3_ has only a slight effect on the photocatalytic performance of ZnO‐V_O_@ZCS_0.20_, while the introduction of p‐BQ significantly inhibits the extraction of U(VI). This observation indicates that ·O_2_^−^ radicals play a pivotal role in the photocatalytic reduction of U(VI).

Furthermore, the catalytic activity was moderately inhibited by the addition of IPA and AA, which can be attributed to the dual formation pathways of ·O_2_^−^ radicals. The one pathway involves the direct reduction of O_2_ by photogenerated *e^−^* (Equation 2), while the other pathway proceeds through the conversion of ·OH radicals (Equations 3 and 4).^[^ ^47^ ^]^

(2) $$O_{2}+e^{-}\to \cdot O_{2}^{-}$$

(3) $$H_{2}O+h^{+}\to \cdot \mathrm{OH}+H^{+}$$

(4) $$4\cdot \mathrm{OH}+e^{-}\to \cdot O_{2}^{-}+2H_{2}O$$

To confirm the origin of ·O_2_^−^ radicals, we conducted controlled experiments by purging the reaction system with Ar to eliminate dissolved O_2_. As shown in Figure S24 (Supporting Information), the photocatalytic U extraction efficiency of ZnO‐V_O_@ZCS_0.20_ was not significantly compromised under an Ar atmosphere, suggesting that ·O_2_^−^ radicals are not exclusively generated through the reduction of O_2_ by photogenerated *e^−^*, but are partially derived from ·OH radical conversion.

EPR spectroscopy was employed to identify the reactive species generated during photocatalysis. As shown in Figure S25 (Supporting information), the signal intensity of TEMO ‐ *h* ^+^ adducts decreased sinificantly under light compared to dark conditions. This decrease indicates that the catalyst generated a large amount of *h* ^+^ and *e* ^‐^ under illumination, and the generated *h* ^+^ oxidized TEMPO to form TEMPO^+^, leading to a reduction in TEMPO ‐ *h* ^+^. Distinct signals corresponding to DMPO‐·OH and DMPO‐·O_2_^−^ adducts are observed under light irradiation, while negligible signals were detected in the dark (Figure 6f,g). Notably, the intensity of DMPO‐·OH and DMPO‐·O_2_^−^ signals for ZnO‐V_O_@ZCS_0.20_ significantly exceeded those of ZnO‐V_O_, indicating enhanced charge carrier separation and redox capacity through heterojunction formation.^[^ ^48^ ^]^

From the perspective of energy band alignment, the more negative valence band (VB) position of ZCS compared to ZnO‐V_O_ suggests superior reducing activity. In a conventional type II heterojunction configuration, *e* ^−^ transfer from ZCS to ZnO‐V_O_ would result in reduced reducing activity and weaker DMPO‐·O_2_^−^ signals. However, our EPR results demonstrate the opposite trend, providing compelling evidence for the formation of an S‐scheme heterojunction rather than a type II heterojunction.^[^ ^49^ ^]^ In this configuration, the recombination of weaker redox *e* ^−^ and *h* ^+^ at the heterojunction interface through the S‐scheme pathway effectively suppresses charge recombination within individual semiconductors, thereby enhancing the overall redox capability of the heterojunction system.

The photocatalytic mechanism of ZnO‐V_O_@ZCS can be summarized as follows (Figure 6h): Upon intimate contact between ZnO‐V_O_ and ZCS, *e* ^−^ spontaneously transfer from ZCS to ZnO‐V_O_ due to the higher Fermi level (*E*_f_) of ZCS, continuing until *E*_f_ alignment is achieved. This *e^−^* redistribution creates an IEF directed from ZCS to ZnO‐V_O_, accompanied by band bending at the interface.^[^ ^43^ ^]^ Under light irradiation, photon absorption promotes *e^−^* from the VB to the CB of both semiconductors. Driven by IEF and mediated by V_O_, *e^−^* in the CB of ZnO‐V_O_ migrate to the VB of ZCS, where they recombine with *h* ^+^ through the S‐scheme pathway. This charge transfer mechanism preserves redox‐active *e* ^−^ in the CB of ZCS and *h^+^* in the VB of ZnO‐V_O_, resulting in enhanced oxidation and reduction potentials that accelerate the photocatalytic reaction. The retained photogenerated *e* ^−^ and generated ·O_2_^−^ radicals collectively contribute to the reduction of U(VI) to UO_2_. Simultaneously, photogenerated *h* ^+^, ·O_2_^−^, and ·OH radicals oxidize water molecules or organic pollutants to O_2_ and benign products, respectively. The proposed reaction pathways are described by Equations (5)‐(10):

(5) $$\mathrm{ZnO}-V_{O}@\mathrm{ZCS}+h\nu \to h^{+}+e^{-}$$

(6) $$O_{2}+e^{-}\to \cdot O_{2}^{-}$$

(7) $$H_{2}O+h^{+}\to \cdot \mathrm{OH}+H^{+}$$

(8) $$4\mathrm{OH}+e^{-}\to \cdot O_{2}^{-}+2H_{2}O$$

(9) $$e^{-}/O_{2}^{-}+{\mathrm{UO}}_{2}^{2+}\to UO_{2}\left(s\right)+O_{2}$$

(10) $$\mathrm{Dyes}+h^{+}/\cdot O_{2}^{-}/\cdot \mathrm{OH}\to \mathrm{harmless}\;\mathrm{products}$$

## 3 Conclusion

In this study, we developed a V_O_‐mediated S‐scheme heterojunction photocatalyst, ZnO‐V_O_@ZCS, for efficient photocatalytic reduction of U(VI). The formation of the S‐scheme heterojunction was unequivocally confirmed through comprehensive characterization techniques, including XPS, in situ XPS, KPFM, fs‐TAS, and EPR spectroscopy, complemented by DFT theoretical calculations. The synergistic effect of enhanced internal electric fields and surface V_O_ facilitates efficient separation and migration of photogenerated charge carriers, enabling rapid U(VI) extraction from nuclear wastewater without sacrificial agents. Remarkably, the optimized ZnO‐V_O_@ZCS_0.20_ photocatalyst achieved 99.10% U(VI) removal within 10 min at pH 4, with an exceptional enrichment capacity of 1024.30 mg g^−1^ in 15 min. The material demonstrated excellent U(VI) enrichment performance across a wide range of conditions, including varying pH levels, coexisting ions/dyes, natural sunlight irradiation, and diverse U‐containing wastewater matrices. This work provides fundamental insights for designing cost‐effective and versatile photocatalysts for practical U recovery applications.

## Conflict of Interest

The authors declare no conflict of interest.

## Supporting information



Supporting Information

## Data Availability

The data that support the findings of this study are available from the corresponding author upon reasonable request.
