# Supplementary material for: Enriched Oxygen Vacancy‐Mediated Efficient Charge Transfer in S‐Scheme ZnO‐VO@Zn0.5Cd0.5S Heterojunction for Rapid U(VI) Removal From Nuclear Wastewater
Source: Adv Sci (Weinh). 2025 Sep 12;12(43):e12163. doi: 10.1002/advs.202512163 (PMC12631871; doi:10.1002/advs.202512163)
Supplement: Supplementary file 1 — Supporting Information [file ADVS-12-e12163-s001.docx]

**Supporting information**

**Enriched Oxygen Vacancy-Mediated Efficient Charge Transfer in S-scheme ZnO-V_O_@Zn_0.5_Cd_0.5_S Heterojunction for Rapid U(VI) Removal from Nuclear Wastewater**

Cailing Liu^1^, Xiaohui Ren^3*^, Smruti Ranjan Sahoo^4^, Artem Kuklin^4^, Chen Yao^2^, Hans Agren^4, 5*^, and Ye Zhang^1,2*^

1. School of Resource, environment and safety engineering, University of South China, Hengyang 421001, China

**E-mail:** yezhang@usc.edu.cn

2. Lab of Optoelectronic Technology for Low Dimensional Nanomaterials, School of Chemistry and Chemical Engineering, University of South China, Hengyang 421001, China.

3. The State Key Laboratory of Refractories and Metallurgy, Key Laboratory for Ferous Metalurgy and Resources Utilization of Ministry of Education & Hubei Provincial Key Laboratory for New Processes of Ironmaking and Steel making, Faculty of Materials, Wuhan University of Science and Technology, Wuhan 430081, P. R. China.

**E-mail:** xhren@wust.edu.cn

4. Department of Physics and Astronomy, Uppsala University, Box 516, Uppsala SE-751 20, Sweden

E-mail: hans.agren@physics.uu.se

5. Faculty of Chemistry, Wroclaw University of Science and Technology, Wyb. Wyspianskiego 27, PL-50370 Wroclaw, Poland

†: These authors contribute equally to this work.

**Experimental Section**

*Chemical Materials*: Anhydrous zinc chloride (ZnCl_2_), polyethylene glycol 600 (PEG600), and acetic acid dihydrate (CH_3_COO)_2_Zn·2H_2_O) were purchased from Shanghai Sinopharm Chemical Reagent Co., Ltd. Ammonia (NH_3_.H_2_O), sodium sulfide hydrate (Na_2_S.9H_2_O) were obtained from Shanghai Titan Scientific Co., Ltd. (CH_3_COO)_2_Cd·2H_2_O, C_2_H_5_NS (TAA) was purchased from Shanghai Aladdin Biochemical Technology Co., Ltd. Arsenazo III and uranyl nitrate hexahydrate ((UO_2_)_2_NO_3_.6H_2_O) were supplied by Shanghai Macklin Biochemical Technology Co., Ltd. All chemicals were employed as received without additional purification.

*Synthesis of ZnO-V_O_*: ZnO-V_O_ was synthesized following a modified procedure adapted from previous work.^[1]^ Specifically, 2.11 g of ZnCl_2_ and 0.9 g of PEG600 were dissolved in 375 mL of deionized water under vigorous stirring. Then, the mixture was sonicated in a water bath for 20 min while adjusting the pH to 10 using NH_3_·H_2_O. The resulting suspension was then transferred to an oil bath maintained at 80 °C and stirred continuously for 21 h. After cooling, the precipitate was washed sequentially with distilled water and ethanol, dried at 40 °C overnight, and manually ground using a mortar to yield powdered ZnO. Final activation was achieved by annealing the powder in a tube furnace at 350 °C for 3 h under atmospheric conditions to generate oxygen-deficient ZnO (ZnO-V_O_).

*Synthesis of* *ZnO-V_O_@ZCS and ZCS*: ZnO-V_O_@ZCS was synthesized based on previous reports with slight modifications.^[2]^ Briefly, 109.75 mg of zinc acetate dihydrate (Zn (CH_3_COO)_2_·2H_2_O, 0.5 mM) and 154.24 mg of cadmium acetate dihydrate (Cd (CH_3_COO)_2_·2H_2_O, 0.5 mM) were dissolved in 50 mL of deionized water under magnetic stirring for 20 min to achieve homogeneity. The solution pH was adjusted to 10.5 using 1 M NaOH, followed by the addition of 75.3 mg thioacetamide (TAA, 1 mM) and stirring for 20 min to form a bright yellow suspension. Subsequently, 202 mg (2.5 mM) of pre-synthesized ZnO-V_O_ was incorporated into the mixture under vigorous stirring for 2 h. The suspension was transferred to a Teflon-lined autoclave and hydrothermally treated at 180 °C for 16 h. Finally, the resultant product was washed repeatedly with ethanol and deionized water, then vacuum-dried at 40°C overnight. The yellowish powder was designated as ZnO-V_O_@ZCS_X_, where X denotes the molar ratio of ZCS to ZnO-V_O_. For comparative analysis, For the purpose of comparative analysis, ZnO@ZCS_0.20_ was prepared using the same method but with unannealed ZnO, while pristine ZCS was synthesized using an identical protocol, omitting the ZnO-V_O_ component.

*Synthesis of ZnO-V_O_@ZCS/SA Gel Spheres*: ZnO-V_O_@ZCS/SA gel spheres were fabricated through divalent cation-alginate crosslinking. Specifically, 60 mg of ZnO-V_O_@ZCS_0.20_ and 60 mg of sodium alginate (SA) were dispersed in 8 mL deionized water under vigorous stirring for 1 h to achieve homogeneity. The homogeneous suspension was dropwise introduced via a 10 μL pipette into 100 mL of 2 % Sr (NO_3_)_2_ aqueous solution at a fixed height, yielding spherical ZnO-V_O_@ZCS_0.20_/SA hydrogel particles (a 0.2-cm diameter). The gel spheres were allowed to cure overnight to strengthen crosslinking integrity.

*The Photocatalytic Extraction of U(VI)*: Typically, the sample (0.08 g/L) and the U(VI) solution (100 mL, 30 mg/L) were added into a photoreactor, with pH adjustment performed using 0.1 M NaOH or HNO_3_. The solution was stirred in the dark for 2 h to ensure that adsorption-desorption equilibrium was reached. Then, the photocatalytic activity toward U(VI) reduction occurred without any sacrificial agents under simulated sunlight irradiation in the air atmosphere using a 300 W xenon lamp (PerfectLight, PLS-SXE300+). After a reaction for a certain time, 1.50 mL dispersion was collected and filtered through a 0.22 μm membrane filter, and then detected by UV-vis spectrophotometry for U(VI) residual concentration based on the Arsenazo III method at a wavelength of 652 nm. The removal rate and removal capacity of the catalysts were calculated by the following equations, respectively. The reaction rate constant is denoted by k.

$$Removal rate \left( \% \right)={(C}_{0}-C_{t})/C_{0} \times100 \%$$

$$Removal capacity \left( {mg}/{g)=(C_{0}}-C_{t} \right) \times V/m$$

$$k=ln(\frac{C_{t}}{C_{0}}) /t$$

where *C*_0_ is the initial concentration of U(VI) (mg /L), C_t_ is the time concentration of U(VI) (mg/L), *V* (L) is the solution volume, *m* (g) is the catalyst dosage.

In addition, the effects of different pH conditions (pH = 3 ~ 8), different U(VI) solution concentrations (C_0_ = 10 ~ 100 mg/L), co-existing ions (K^+^, Na^+^, Ca^2+^, Mg^2+^, Zn^2+^, Co^2+^, Cu^2+^, Pb^2+^, Cl^-^, Br^-^, SO_4_^2-^ and CO_3_^2-^; C_U(VI)_/C_ions_ = 1:10) and co-existing dyes (Rhodamine B (RHB), crystalline purple (CV)and methylene blue (MB); C_dye_ = 10 mg/L) on the U(VI) photocatalytic extraction performance of the samples were investigated. To test the reusability of the samples, the post-photocatalysis solid was recovered via filtration, subjected to elution in 0.1 M NaHCO_3_ solution for 1.5 h, rinsed with deionized water until neutral pH, and reintroduced into subsequent photocatalytic cycles after being thoroughly dried. (All experiments were performed in triplicate, with results expressed as mean ± standard deviation.)

*Electrochemical Measurements*: The electrochemical tests were implemented on a CHI660C electrochemical workstation (Chenhua Instrument Co., Ltd., Shanghai, China) with a three-electrode system. The photocatalyst-coated indium tin oxide (ITO) electrode served as was used as the working electrode, and platinum electrode and saturated silver chloride electrode were served as the reference electrode of the working electrode, respectively. Mott-Schottky (M-S) analysis, electrochemical impedance spectroscopy (EIS), and photocurrent density measurements were performed in 0.5 M Na_2_SO_4_ electrolyte.

*Material Characterization*: The phase purity was evaluated by X-ray diffraction (TongDa, TD-3500). X-ray photoelectron spectroscopy (XPS) analysis was performed using an Al Kα X-ray source (1.5418 Å, Thermo Fisher Escalab Xi+), with binding energies calibrated against the adventitious C 1s peak at 284.8 eV. Scanning electron microscopy (SEM, Zeiss Gemini 300) and transmission electron microscopy (TEM, JEOL JEM 2100F) were used to record the morphology and microstructural features. Ultraviolet-visible diffuse reflectance spectra (UV-vis DRS) were performed on a Hitachi UV-3900 spectrophotometer. The steady-state photoluminescence (PL) spectra were analyzed by a fluorescence spectrophotometer (Hitachi F-7000). The Edinburgh FLS5 fluorescence spectrophotometer recorded time-resolved photoluminescence spectra (TRPL). The Fourier Transform infrared (FTIR) spectra were investigated using a spectrometer (Thermo Fisher Scientific). The electron paramagnetic resonance (EPR) measurements were recorded on an EPR spectrometer (Bruker, EMXPLUS).

*Computational Methods*: The structural and electronic properties of ZnO, ZnCdS, and their heterojunction interface were investigated using DFT calculations performed with the Vienna Ab initio Simulation Package (VASP).^[3, 4]^ Electron-ion interactions were treated within the projector augmented wave (PAW) framework, while exchange-correlation effects were described using the Perdew-Burke-Ernzerhof (PBE)^[5]^ generalized gradient approximation (GGA). To account for van der Waals (vdW) interactions and achieve accurate structural relaxation, we incorporated Grimme's DFT-D3^[6]^ dispersion correction. This approach is particularly crucial for correctly describing interlayer interactions at the heterojunction interface, as standard GGA tends to overestimate lattice constants and underestimate band gaps due to insufficient treatment of electron-electron correlations.

All calculations employed a plane-wave cutoff energy of 400 eV and gamma-centered k-point meshes ranging from 6×6×1 to 12×12×12, depending on the unit cell dimensions. A vacuum layer of 15 Å was introduced in slab models to prevent spurious interactions between periodic images. Structural optimizations were performed using the conjugate-gradient method until atomic forces converged below 0.01 eV/Å. To accurately describe electronic properties, we complemented standard PBE calculations with hybrid functional approaches (PBE0^[7]^ and HSE06^[8]^) for density of states analysis, as these methods provide more reliable band gap predictions.

The optimized hexagonal wurtzite ZnO structure (space group P63/mc) exhibited lattice parameters consistent with previous experimental and theoretical reports.^[9]^ For ZnCdS, the optimized bulk structure (space group F-43m) displayed lattice constants (*a* = 5.683 Å, *b* = *c* = 5.582 Å, *α* = *β* = *γ* = 90.0 °) intermediate between those of ZnS and CdS,^[10, 11]^ reflecting its face-centered cubic character. The ZnO@ZnCdS heterostructure was modeled using a (110) interface slab containing 168 atoms in a 1×1 unit cell, optimized at the GGA/PBE+DFT-D3 level of theory.


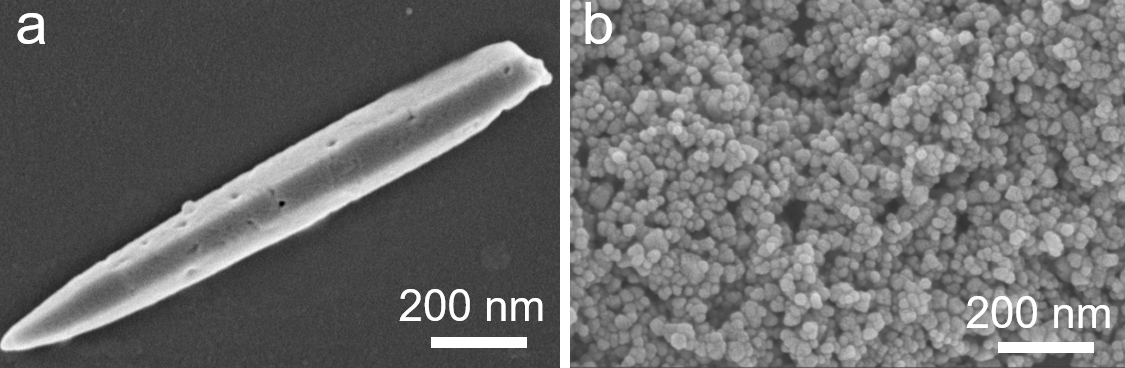


**Fig. S1.** SEM images of (**a**) ZnO-V_O_, (**b**) ZCS.


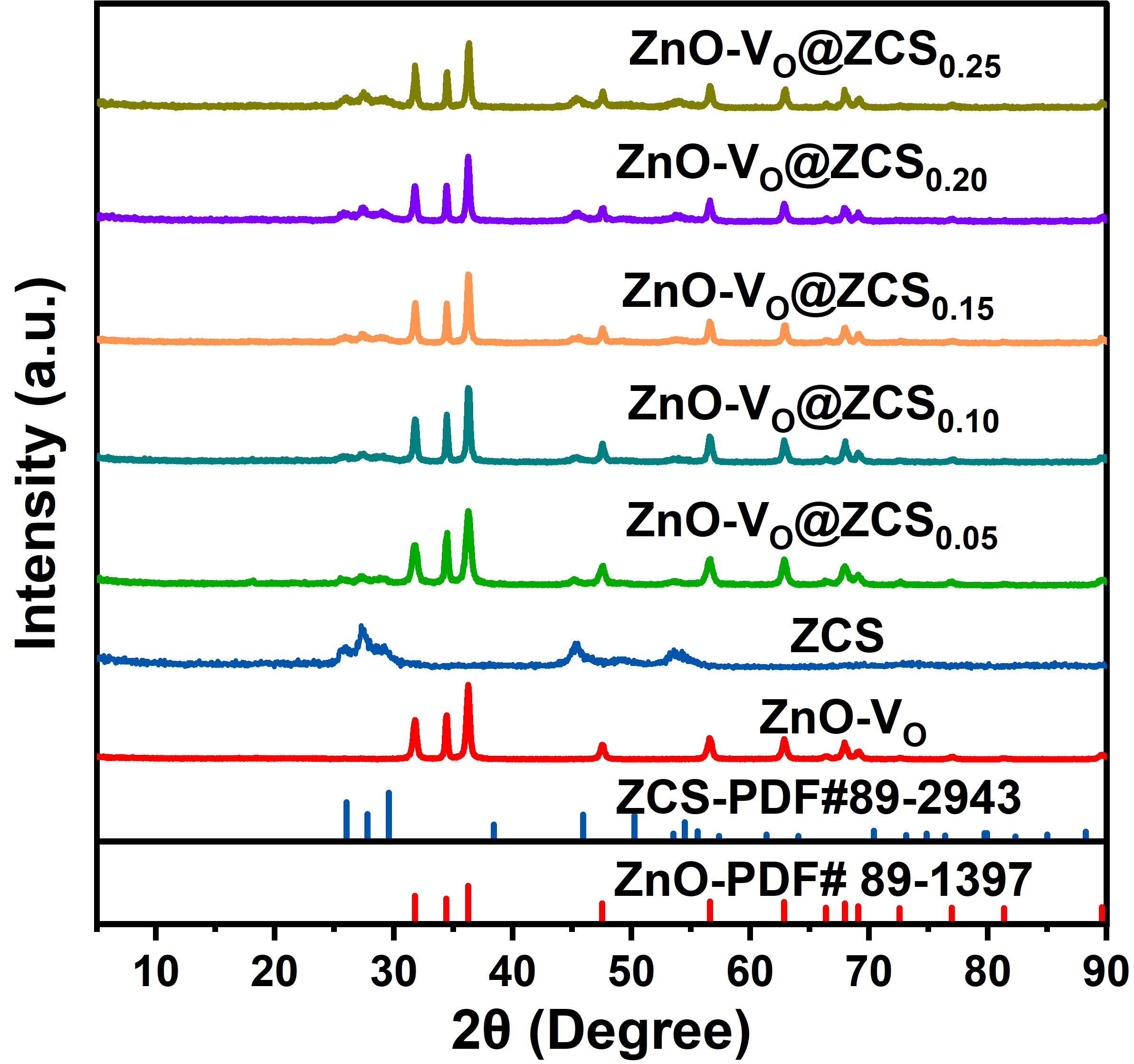


**Fig. S2.**  XRD patterns of different samples.


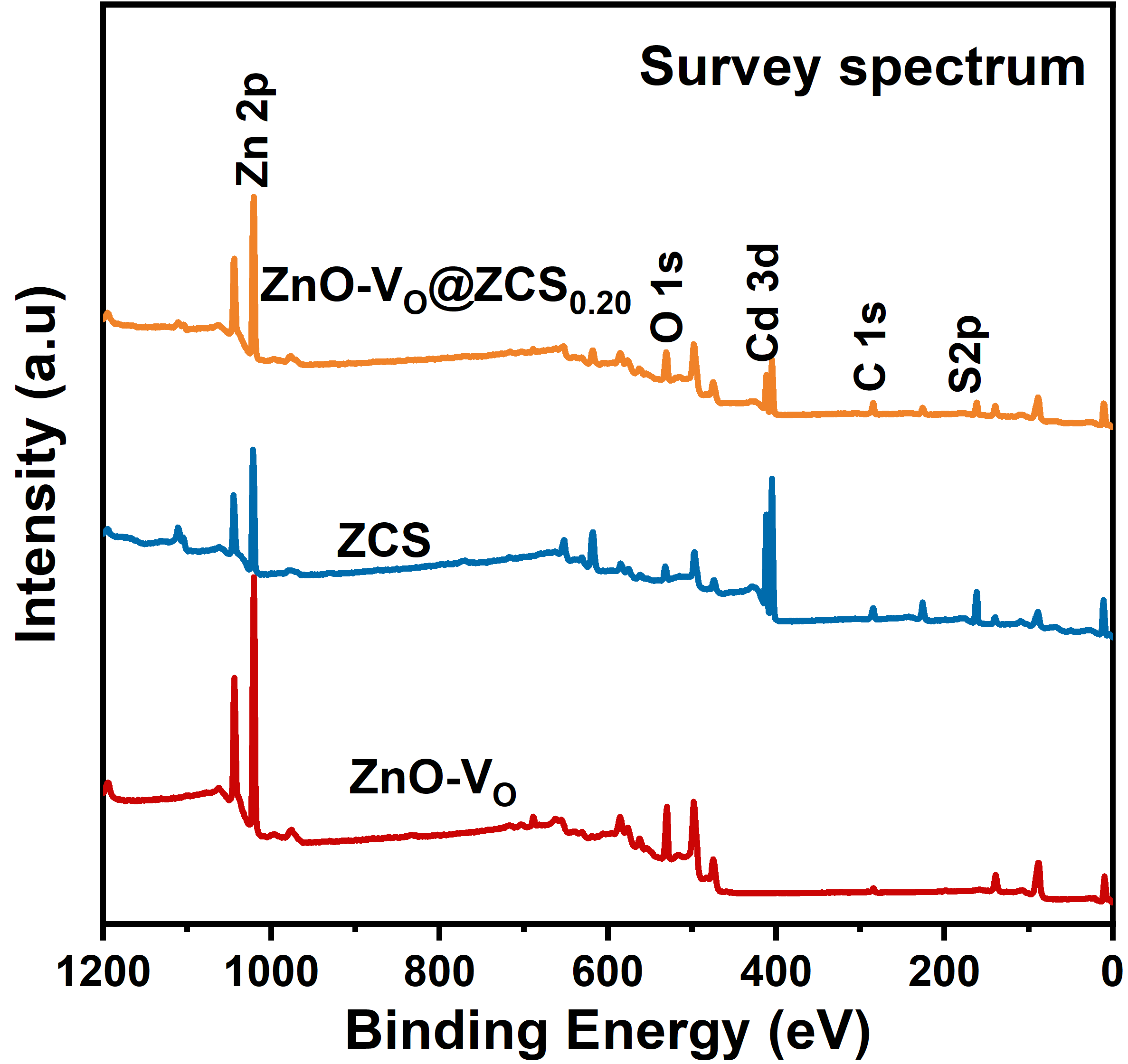


**Fig. S3.** The XPS survey spectra of the ZnO-V_O_, ZCS, and ZnO-V_O_@ZCS_0.20_ samples.

**
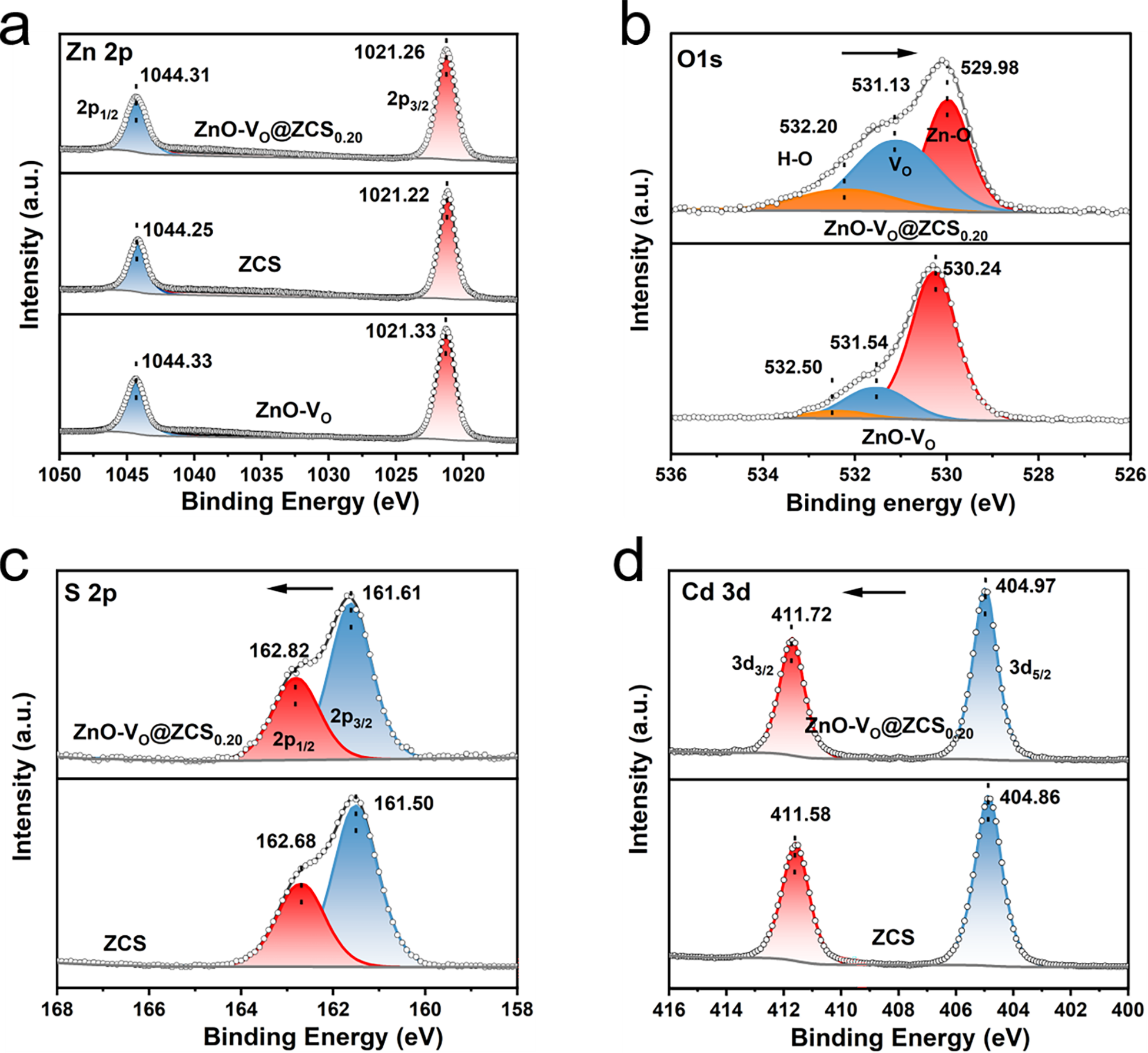
**

**Fig. S4.** High‐resolution XPS spectra of (**a**) Zn 2p, (**b**) O 1s, (**c**) S 2p, and (**d**) Cd 3d in ZnO-V_O_, ZCS, and ZnO-V_O_@ZCS_0.20_ samples.


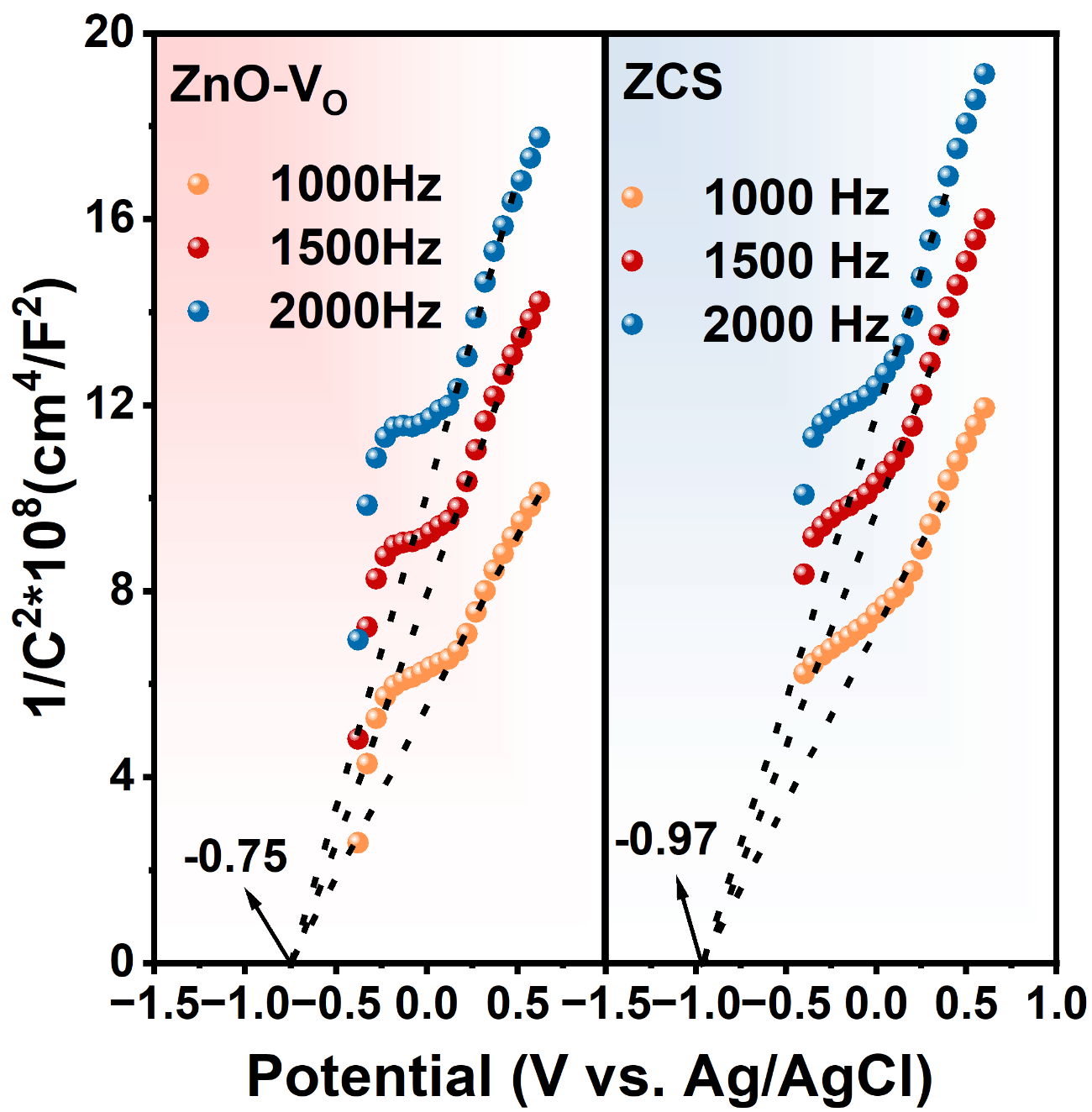


**Fig. S5.** M-S plots of ZnO-V_O_ and ZCS.

**
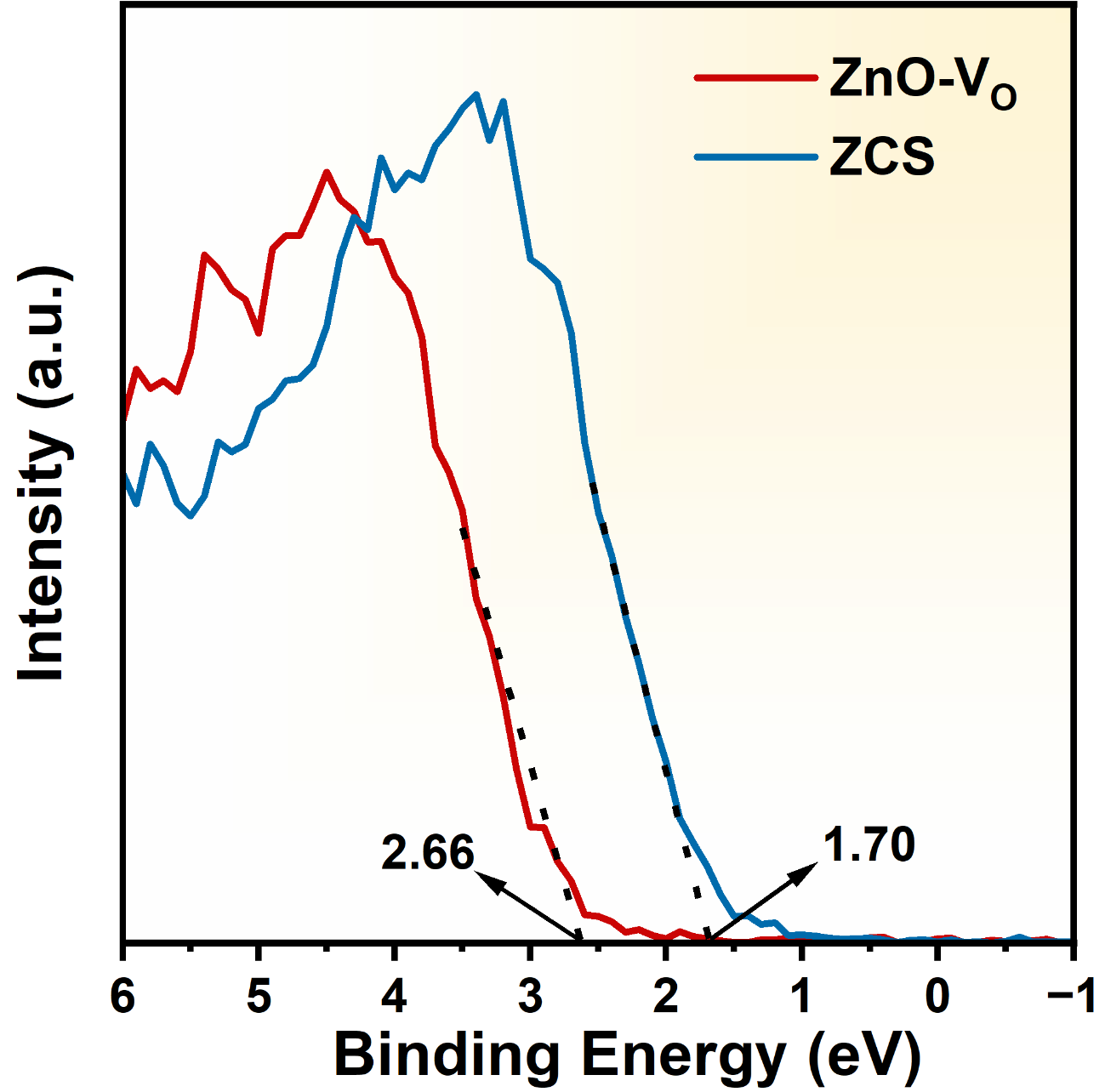
**

**Fig. S6.** Valence band spectrum of ZnO-V_O_ and ZCS.


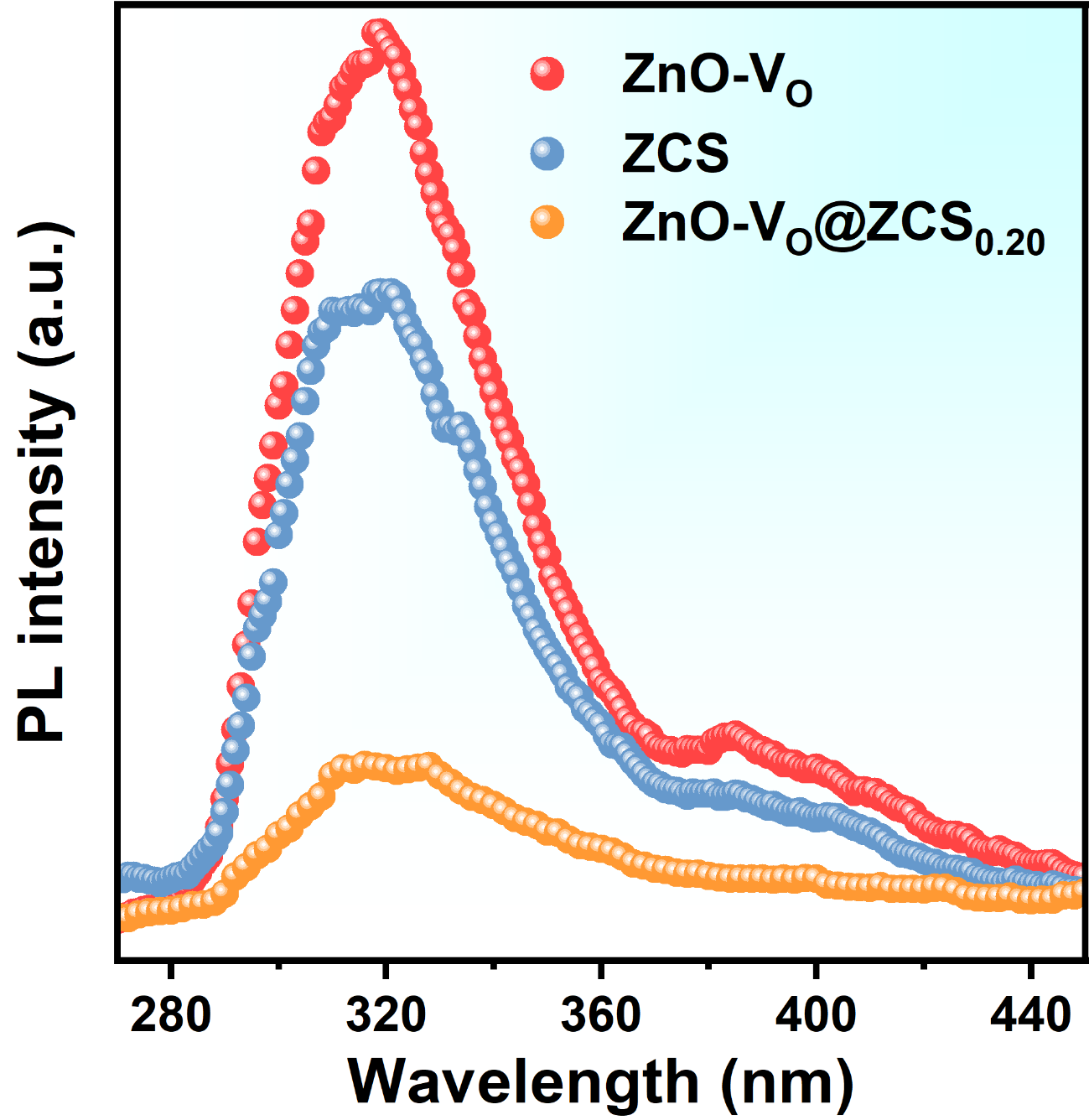


**Fig. S7.** PL spectra of ZnO-V_O_, ZCS, and ZnO-V_O_@ZCS_0.20_.

| **Samples** | **τ_1_ (ns)** | **τ_2_ (ns)** | **Rel_1_%** | **Rel_2_%** | **τ_a_ (ns)** |
| --- | --- | --- | --- | --- | --- |
| **ZnO-V_O_** | 53.34 | 736.53 | 21.57 | 78.43 | 589.17 |
| **ZCS** | 23.72 | 348.68 | 17.46 | 82.54 | 291.94 |
| **ZnO-V_O_@ZCS_0.20_** | 72.09 | 1096.75 | 17.59 | 82.41 | 916.51 |

**Table S1.** The corresponding fitting parameters for the time-resolved PL decay plots of the samples.


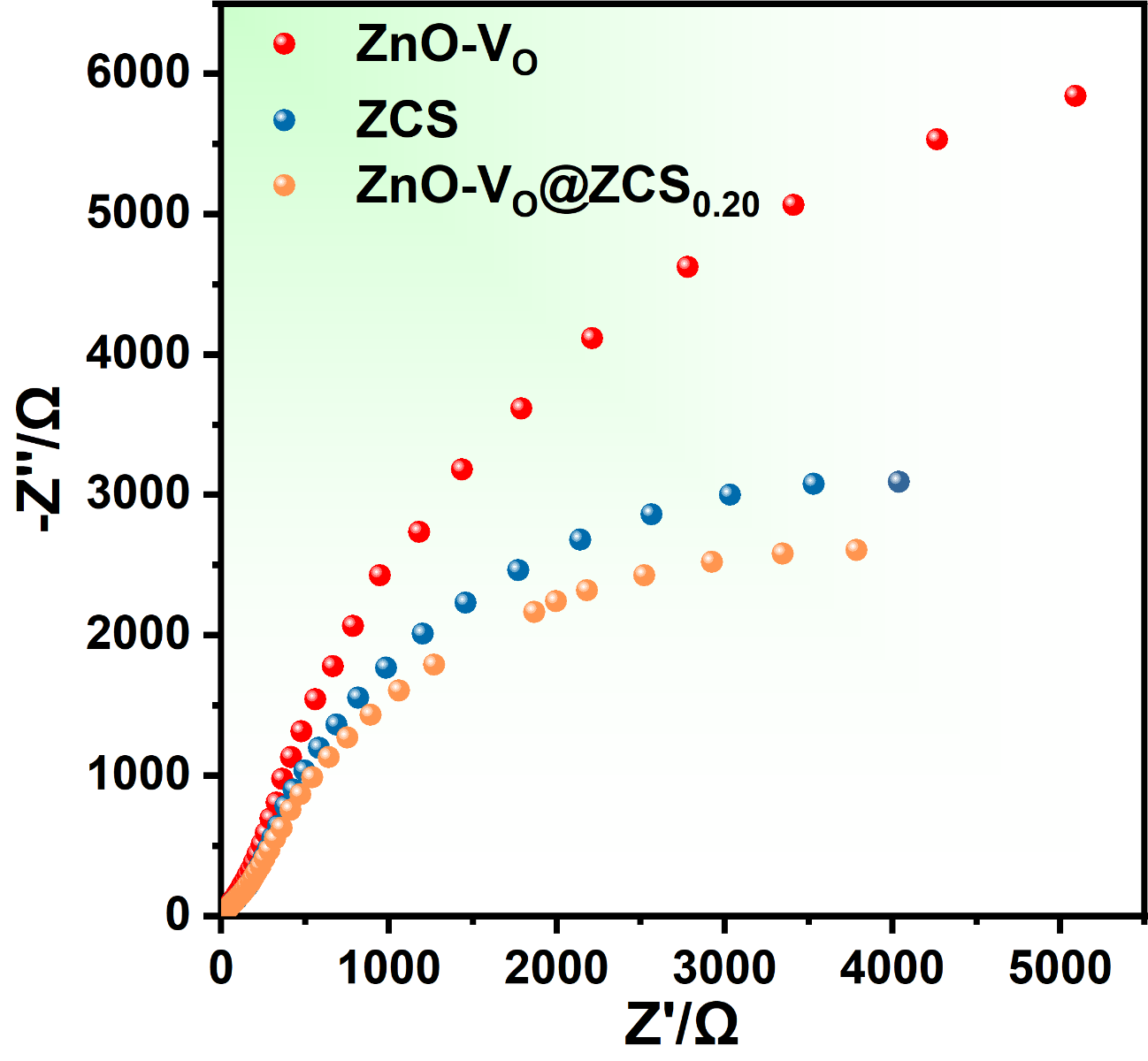


**Fig. S8.** EIS plots of ZnO-V_O_, ZCS, and ZnO-V_O_@ZCS_0.20_.

**
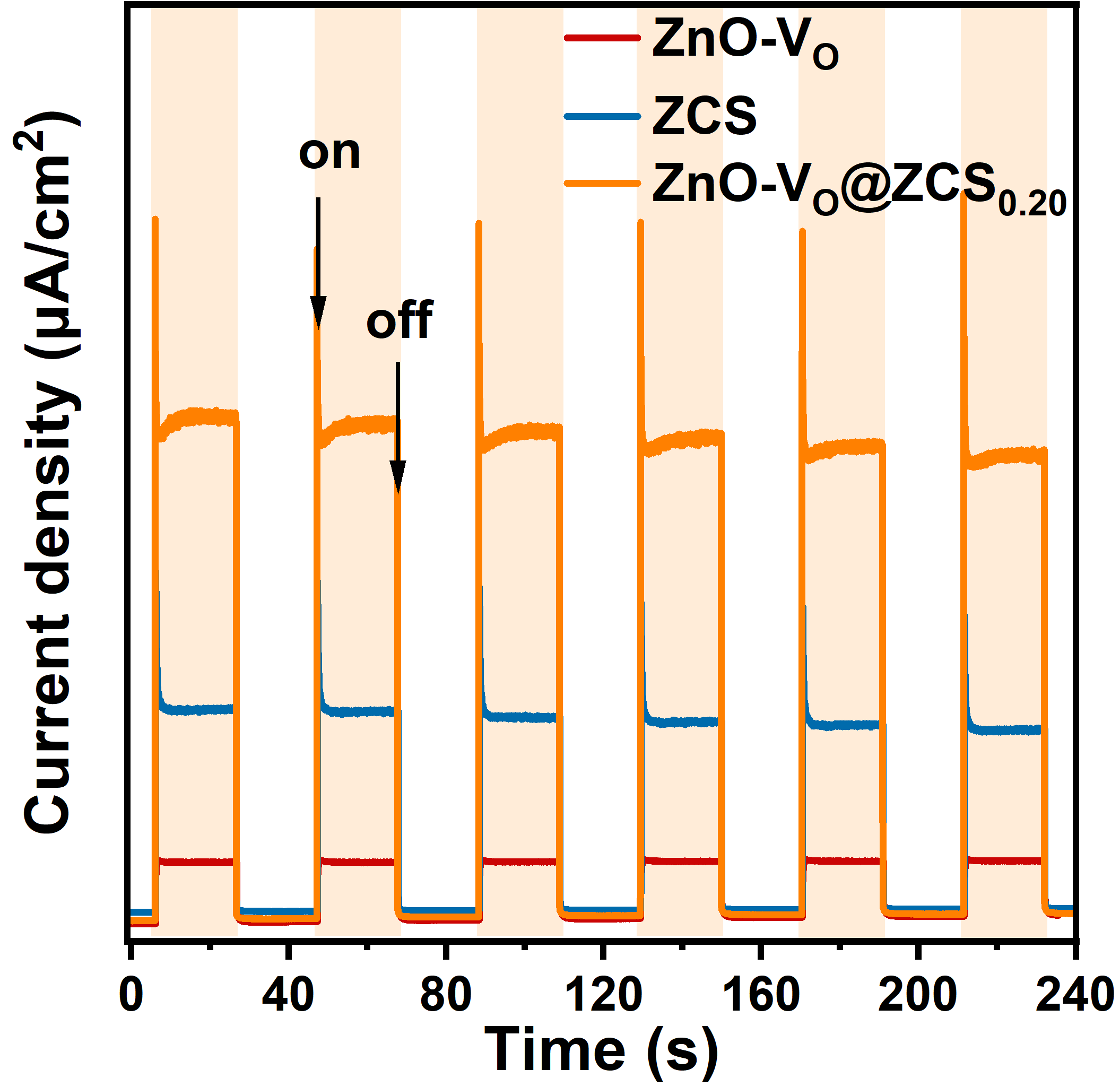
**

**Fig. S9.** Transient photocurrent responses of ZnO-V_O_, ZCS, and ZnO-V_O_@ZCS_0.20_.

**
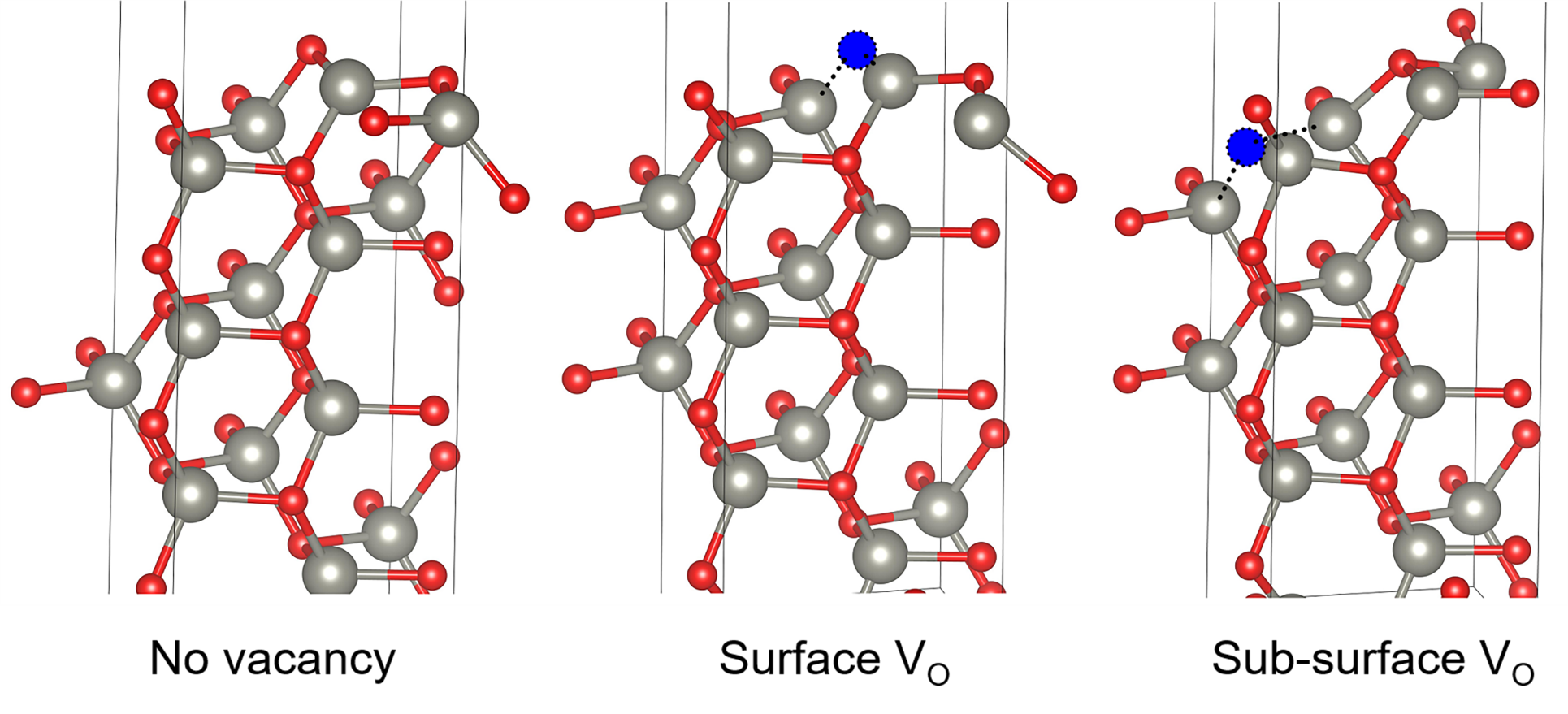
**

**Fig. S10.** Optimized structure for $1\times1$ unit cell surface ZnO slab with and without V_O_. The removed oxygen atom in the ZnO-V_O_ surface slab is shown in blue color as a dotted ball.

**
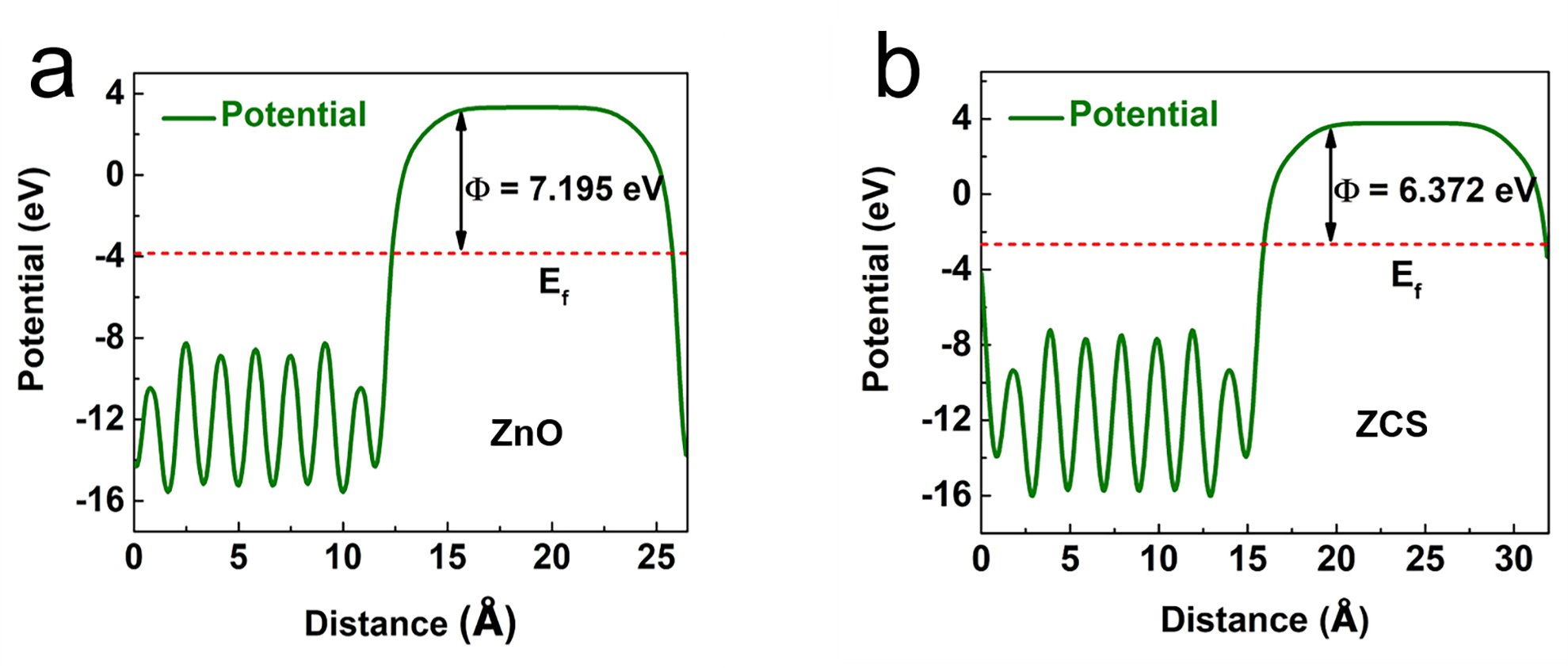
**

**Fig. S11.** Calculated potential energy and work function for (**a**) (110) 1×1 unit cell surface slab of ZnO at the PBE0 level of theory, (**b**) (110) 1×1 unit cell surface slab of ZnCdS at the HSE06 level of theory.

**
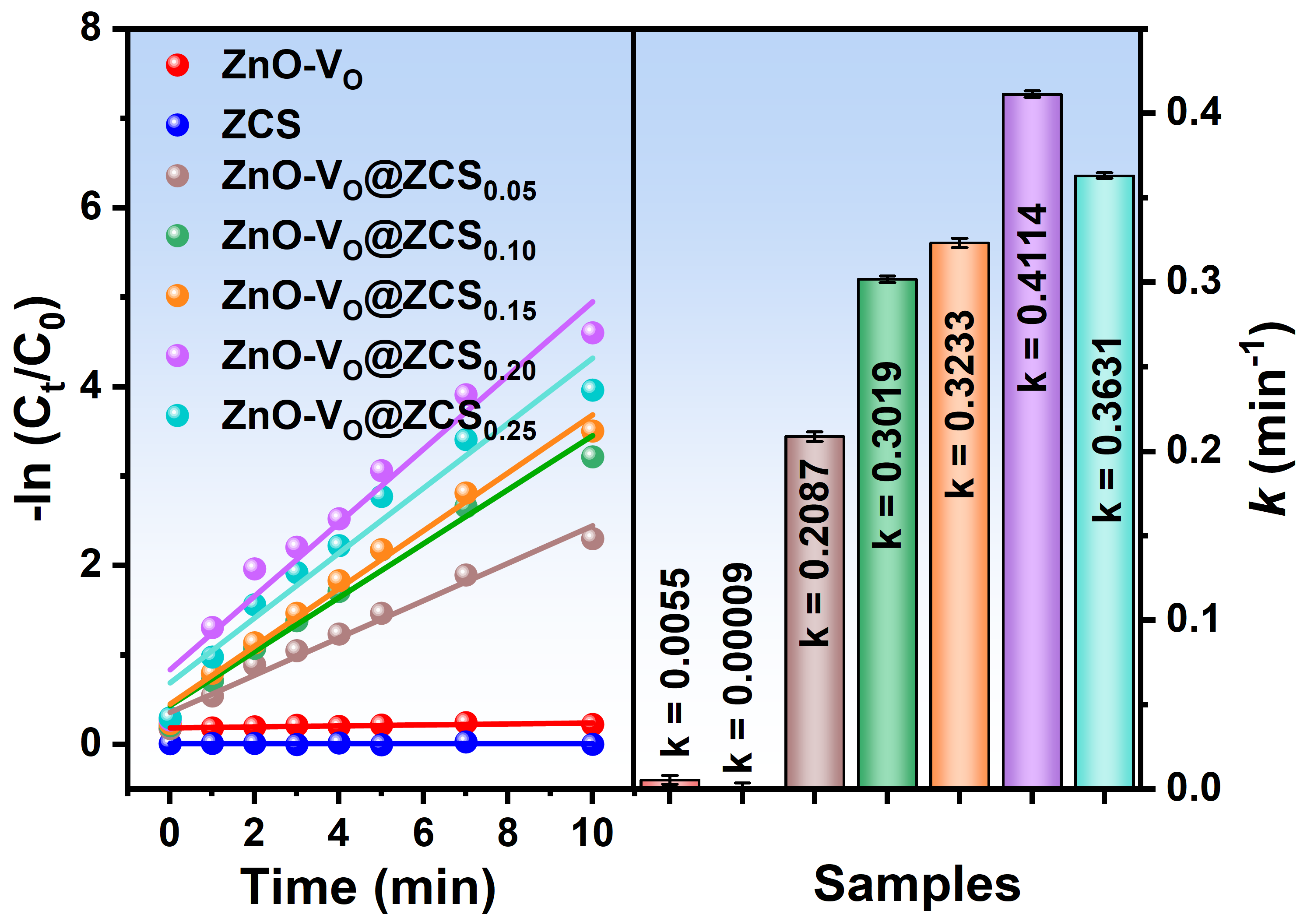
**

**Fig. S12.** The kinetic rate constant (*k*) over different photocatalysts.

**Table S2.** The different catalysts for U(VI) photoreduction in recent literature.

| **Photocatalysts** | **U(VI) Concentration (mg/L)** | **Solid-to-liquid ratio (g/L)** | **Sacrificial agent** | **Time**  **(min)** | **Removal rate (%)** | **Ref.** |
| --- | --- | --- | --- | --- | --- | --- |
| ZnO-V_O_@Zn_0.5_Cd_0.5_S | 30 | 0.08 | none | 10 | 99.1 | **This work** |
| Cu_3_-PA-COF-AA | 20 | 0.05 | none | 390 | 93.6 | ^[12]^ |
| TpTt | 30 | 0.229 | Methanol | 600 | 90 | ^[13]^ |
| DHBQ-TAPP-COF | 60 | 0.05 | none | 180 | >80 | ^[14]^ |
| B-TiO_2_@Co_2_P-500 | 8 | 0.25 | none | 90 | 98 | ^[15]^ |
| Ni-SCN@G | 64 | 0.033 | methanol | 11 | 99.8 | ^[16]^ |
| WO_x_/g-C_3_N_4_ | 20 | 0.4 | benzyl alcohol | 120 | 98.5 | ^[17]^ |
| C_4_N/C_6_N_7_ | 27.6 | 0.5 | none | 60 | 97.2 | ^[18]^ |


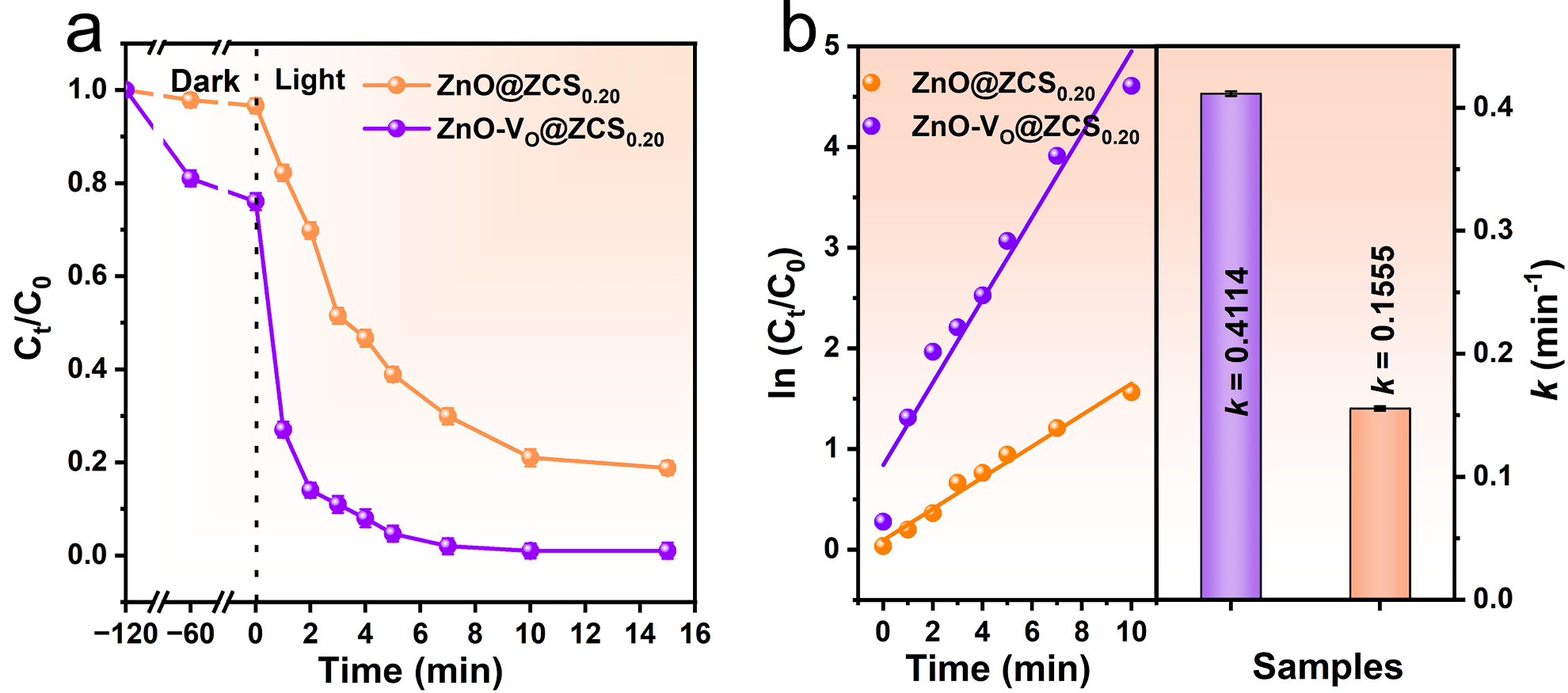


**Fig. S13.** Comparative performance plots of photocatalytic reduction of U(VI) by ZnO-V_O_@ZCS_0.20_ and ZnO@ZCS composites.

**
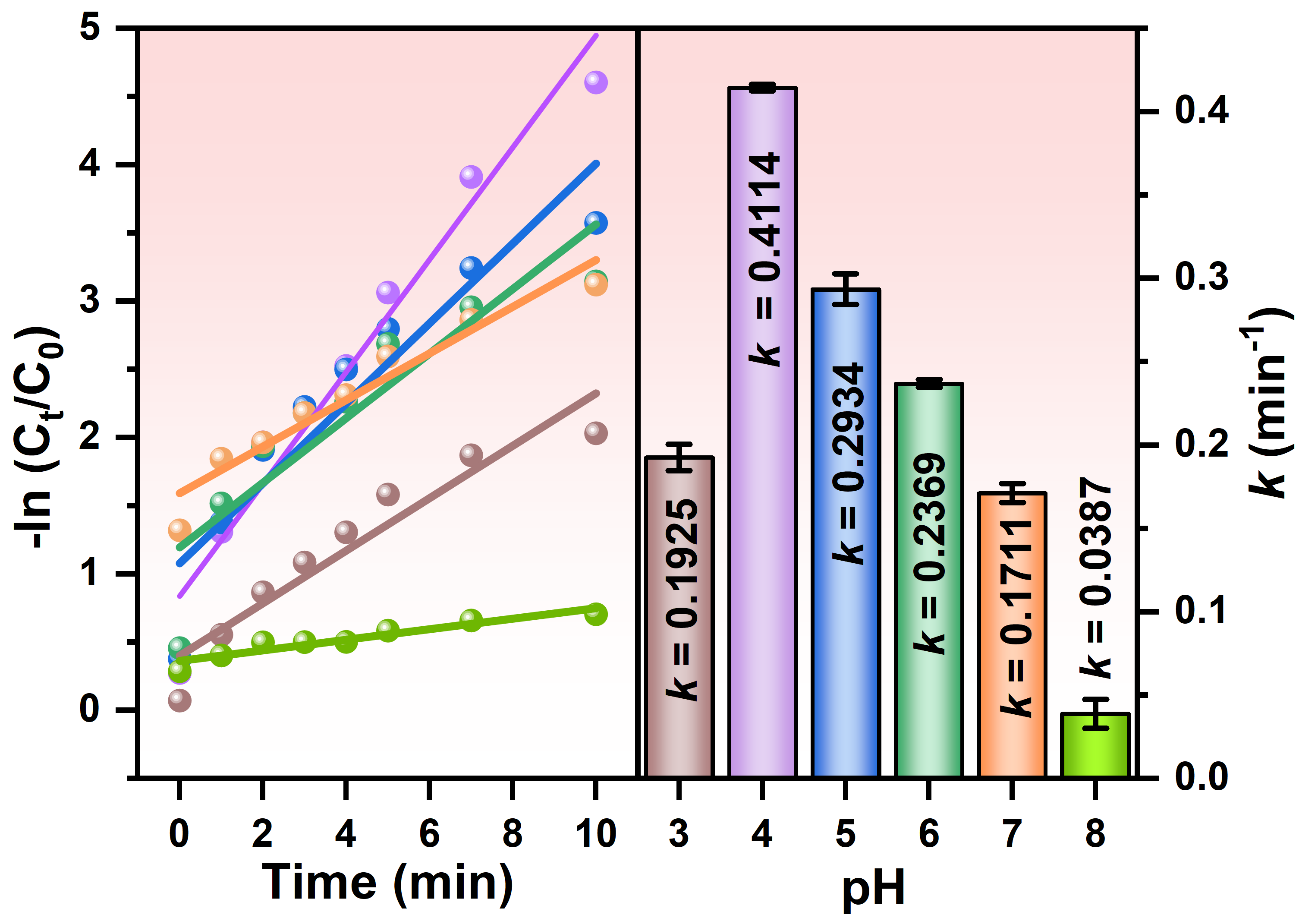
**

**Fig. S14.** The kinetic rate constant (*k*) of ZnO-V_O_@ZCS_0.20_ at different pH conditions.


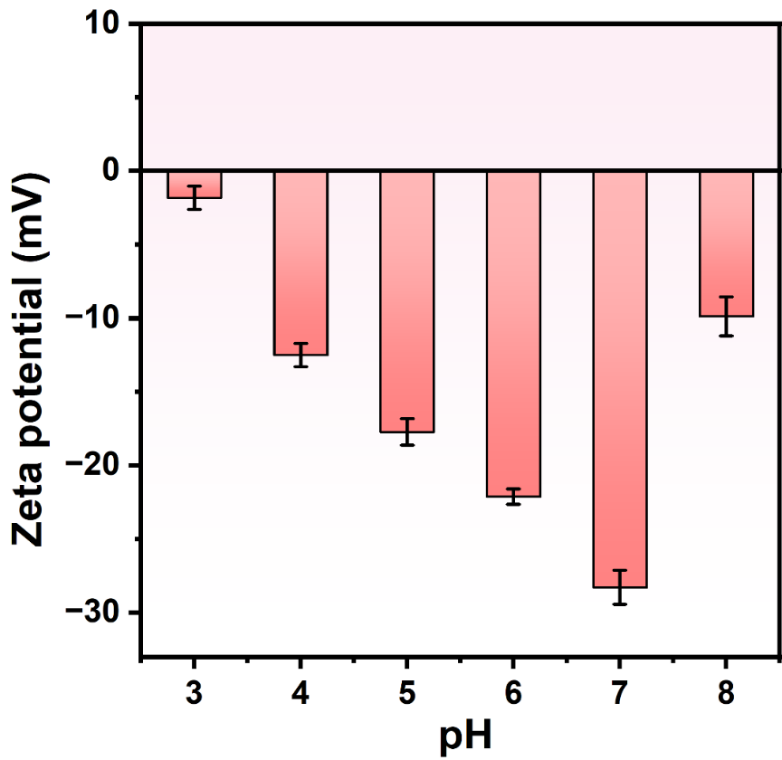


**Fig. S15.** Zeta potential profile of ZnO-V_O_@ZCS_0.20_ as a function of pH value.

**
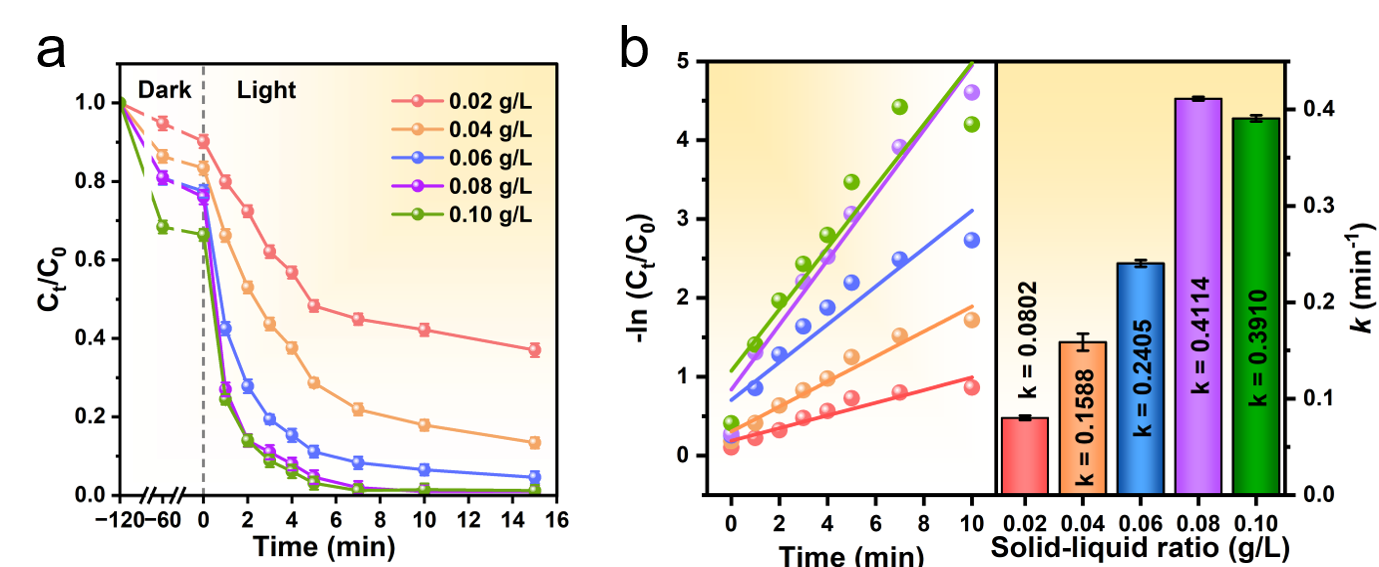
**

**Fig. S16. (a)** The effect of different solid-liquid ratios on the photocatalytic reduction of U(VI) activity and (**b**) the corresponding kinetic rate constant (*k*).

**
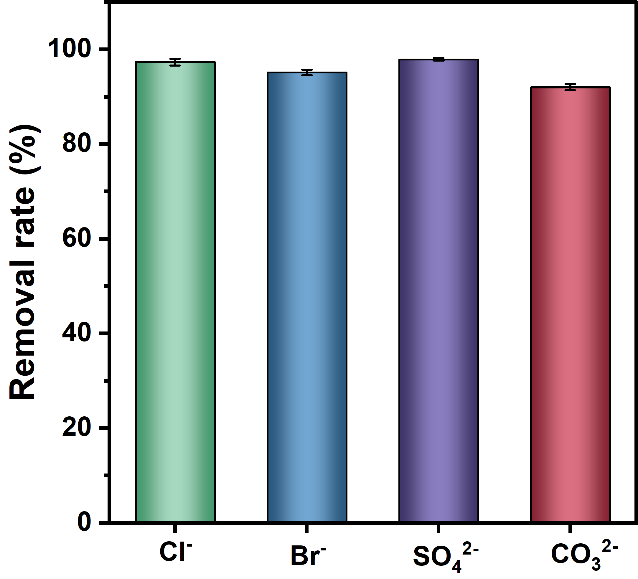
**

**Fig. S17.** The effect of different coexisting anions on the photocatalytic reduction of U(VI) properties of ZnO-V_O_@ZCS_0.20_.


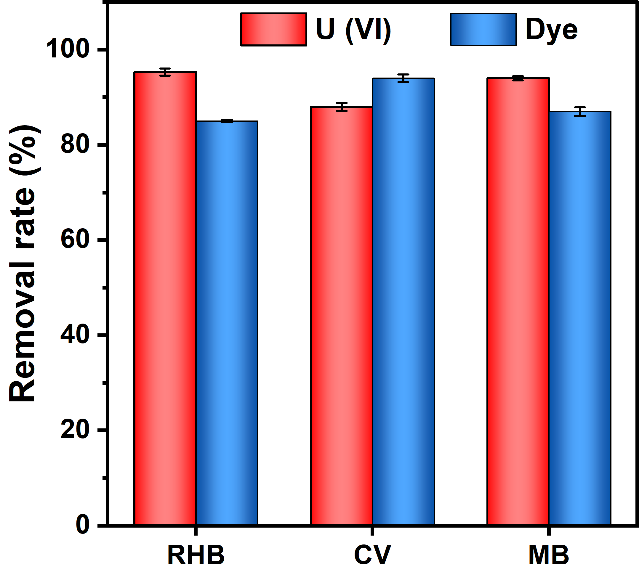


**Fig. S18.** Removal properties of ZnO-V_O_@ZCS_0.20_ for dyes and U(VI) when dyes coexist with U(VI) (experiment conditions: m/V = 0.08 g L^−1^, C_U(VI)_ = 30 mg L^−1^, C_dye_ = 10 mg/L, pH = 4, t = 60 min, T = 298 K).

**Table. S3.** The main component parameters of the uranium tailings wastewater.

| **Parameters** | **U** | **Zn** | **Ca** | **Mg** | **Fe** | **Mn** | **NH^4+^-N** | **Cl^-^** | **NO^3-^** | **SO_4_^2-^** | **COD** | **Conductivity** |
| --- | --- | --- | --- | --- | --- | --- | --- | --- | --- | --- | --- | --- |
|  | (μg/L) | (mg/L) | (mg/L) | (mg/L) | (mg/L) | (mg/L) | (mg/L) | (mg/L) | (mg/L) | (mg/L) | (mg/L) | (μS/cm) |
| **Content** | 497.68 | 6.90 | 418.20 | 44.67 | 0.04 | 11.98 | 9.85 | 108.97 | 384.88 | 2047.98 | 35 | 3513 |


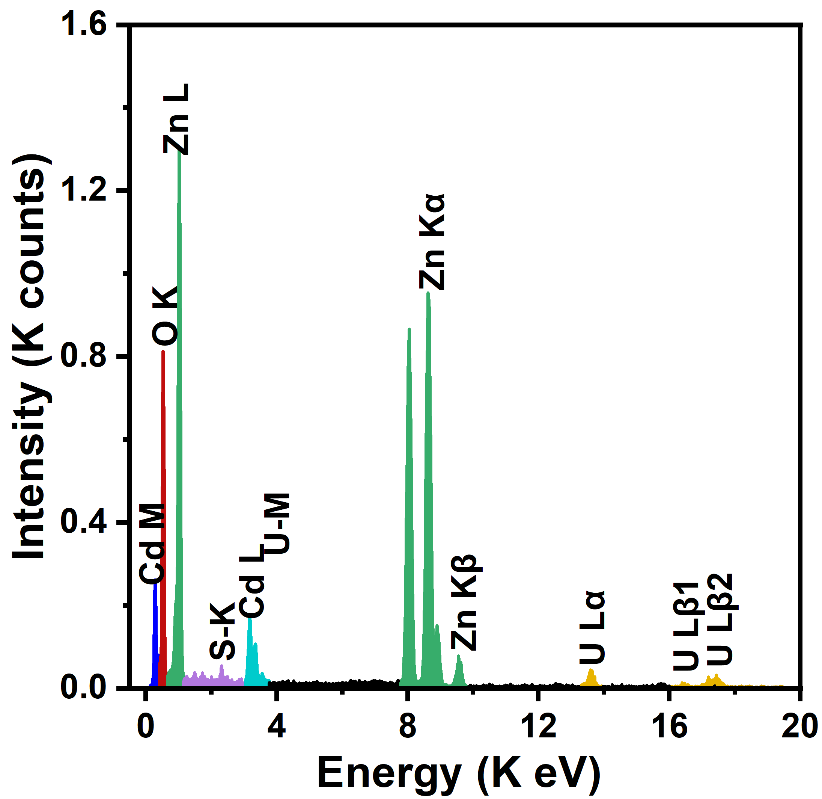


**Fig. S19.** EDS spectrum of the ZnO-V_O_@ZCS_0.20_ after photocatalysis under the TEM model.


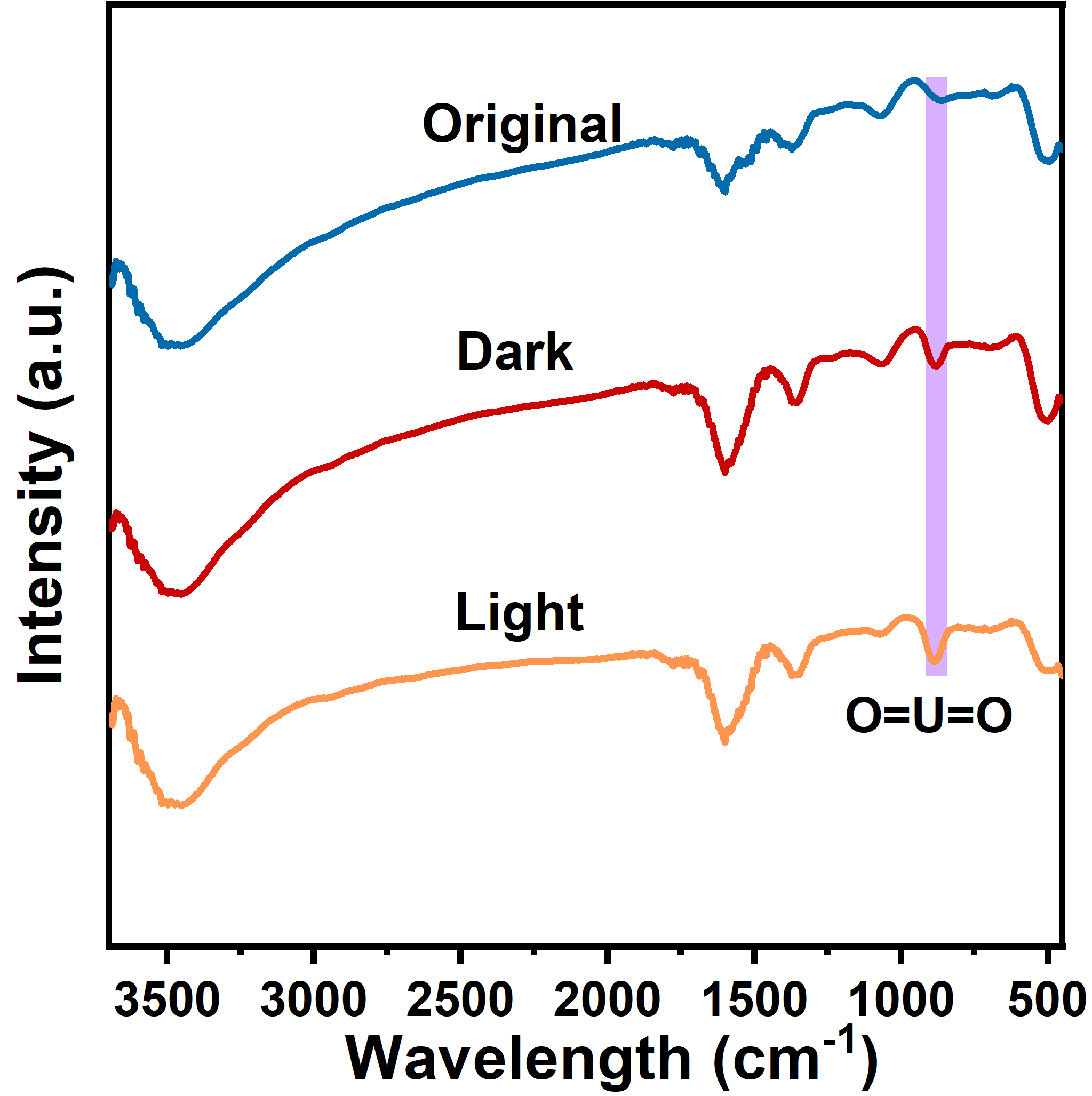


**Fig. S20.** The FTIR Spectra of ZnO-V_O_@ZCS_0.20_ before and after U extraction.


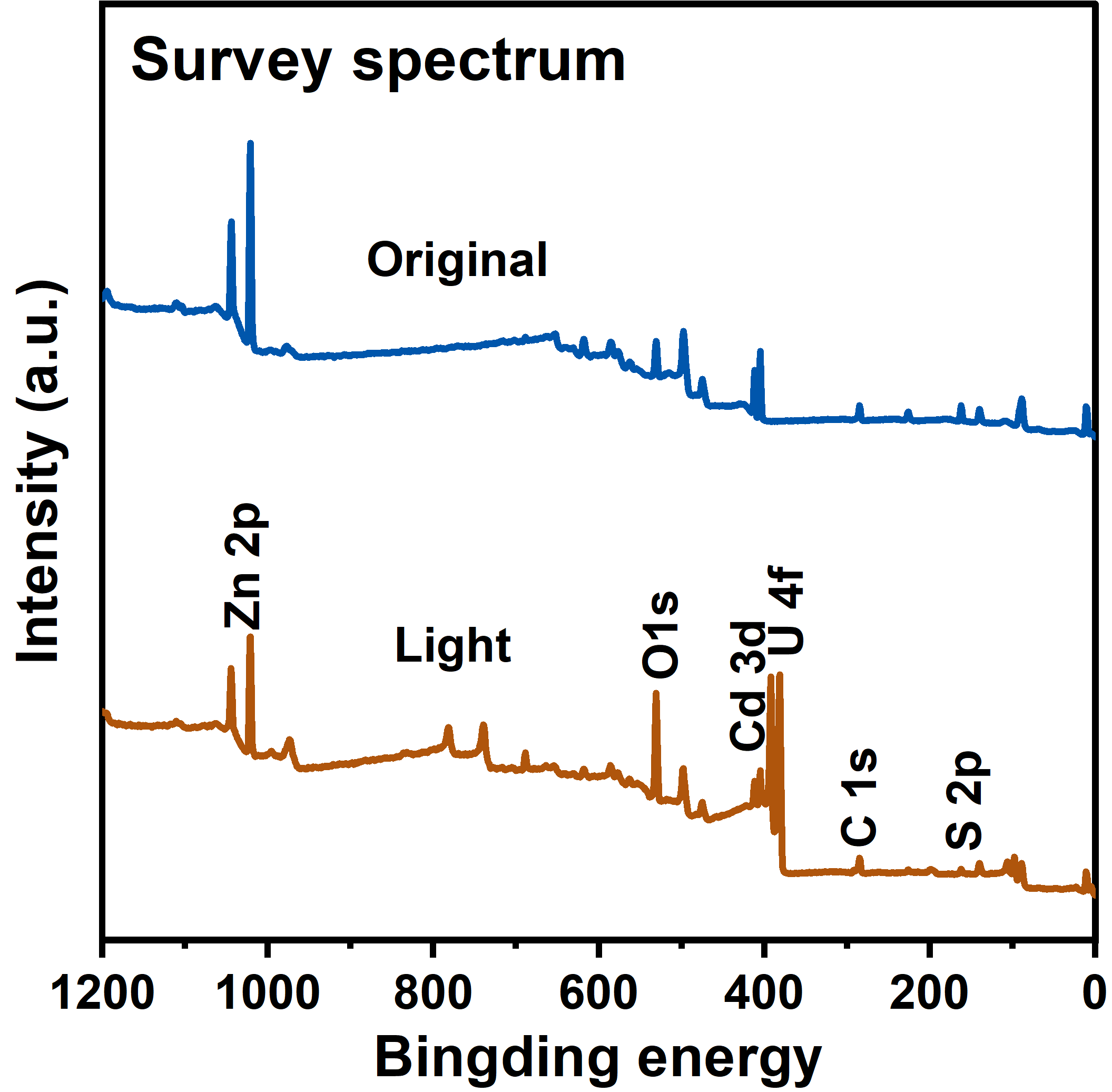


**Fig. S21.** XPS survey spectra before and after photocatalysis of ZnO-V_O_@ZCS_0.20_.


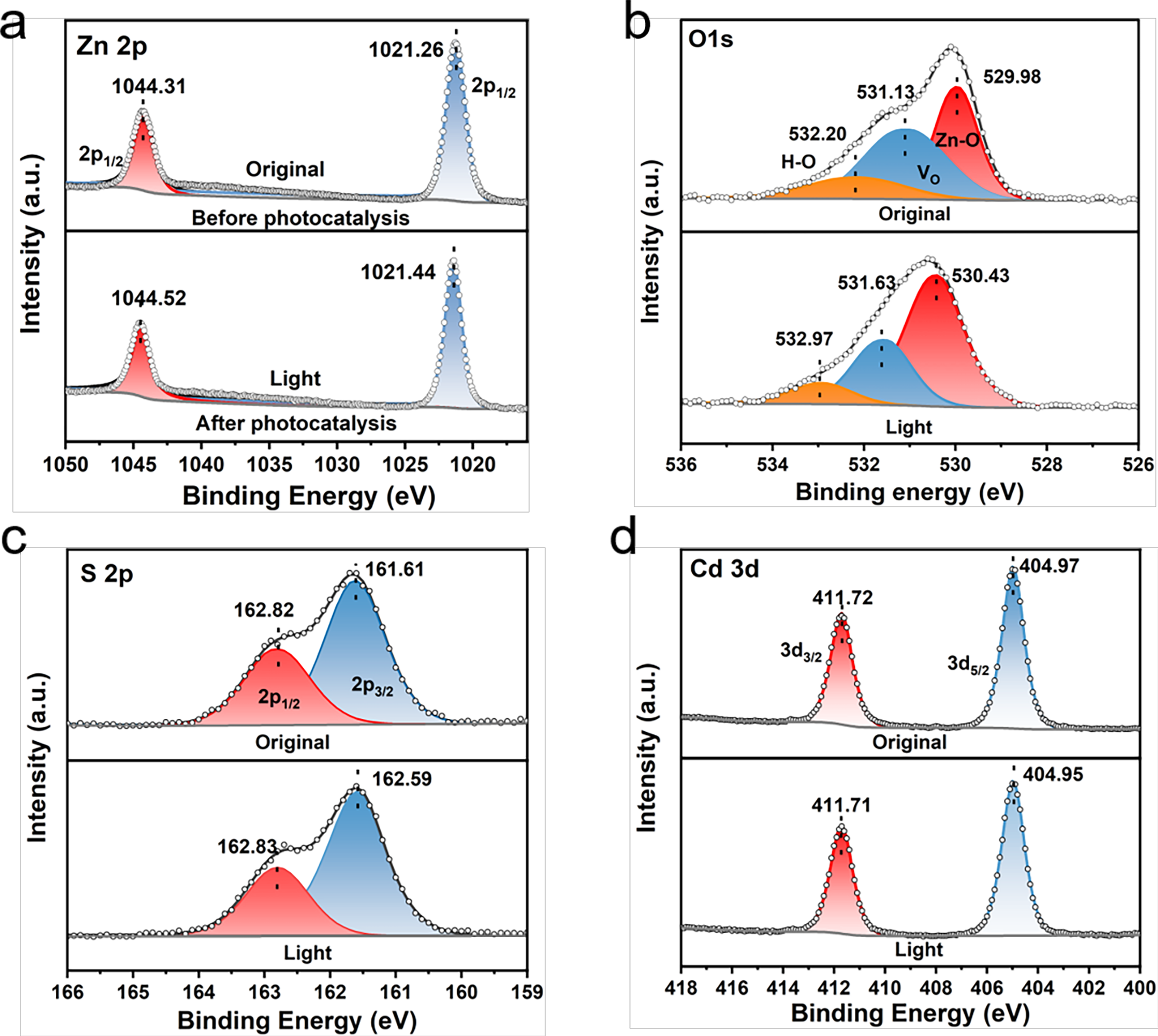


**Fig. S22.** (**a**) Zn2p, (**b**) O1s, (**c**) S 2p, and (**d**) Cd 3d XPS spectrum before and after photocatalysis of ZnO-V_O_@ZCS_0.20_.


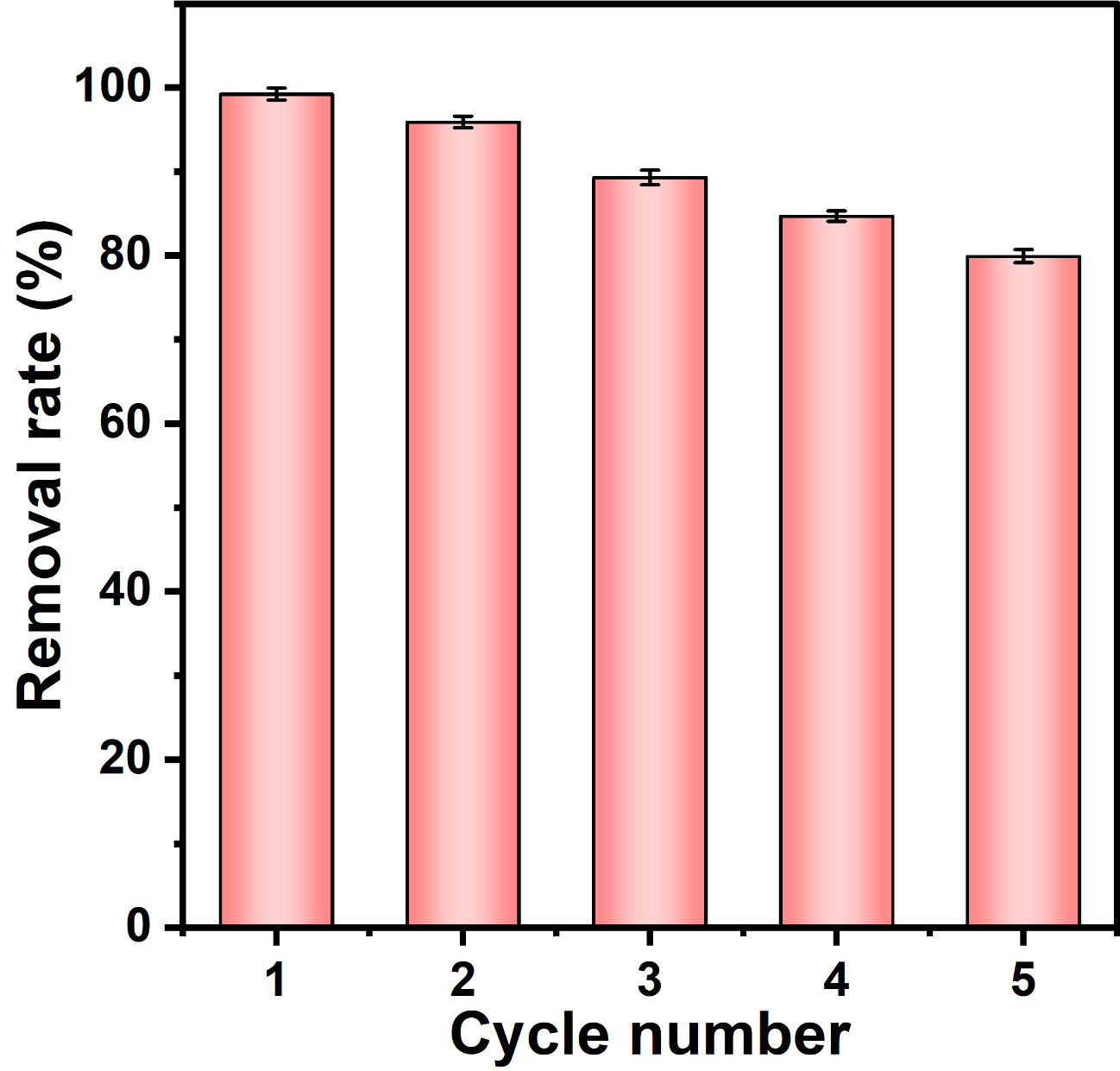


**Fig. S23.** Recyclability of ZnO-V_O_@ZCS_0.20_ for photocatalytic U extraction.


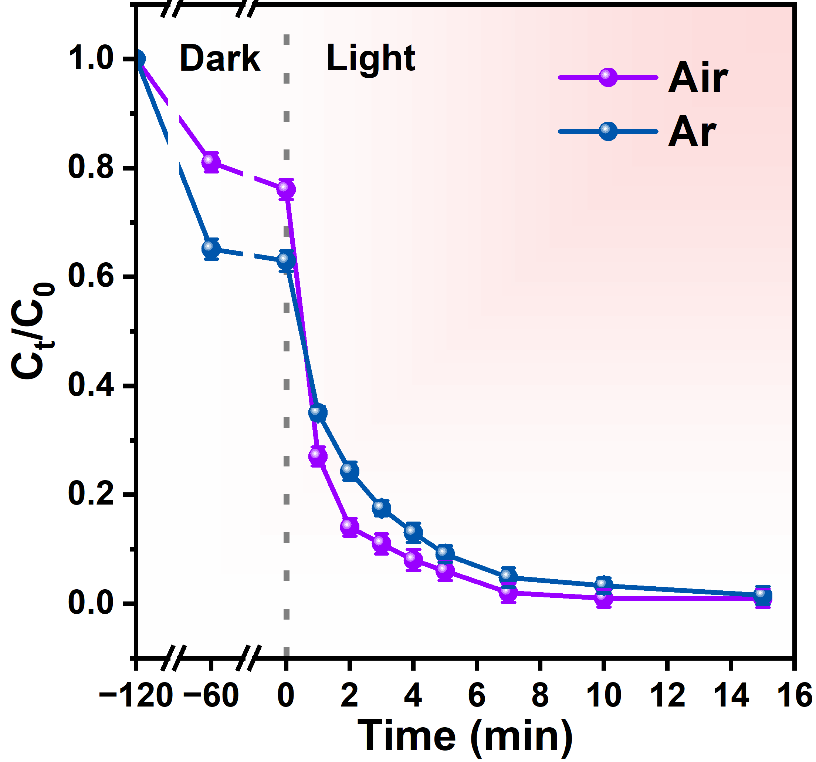


**Fig. S24.** Effect of Ar injection in the photocatalytic system on removing U(VI) by ZnO-V_O_@ZCS_0.20_.


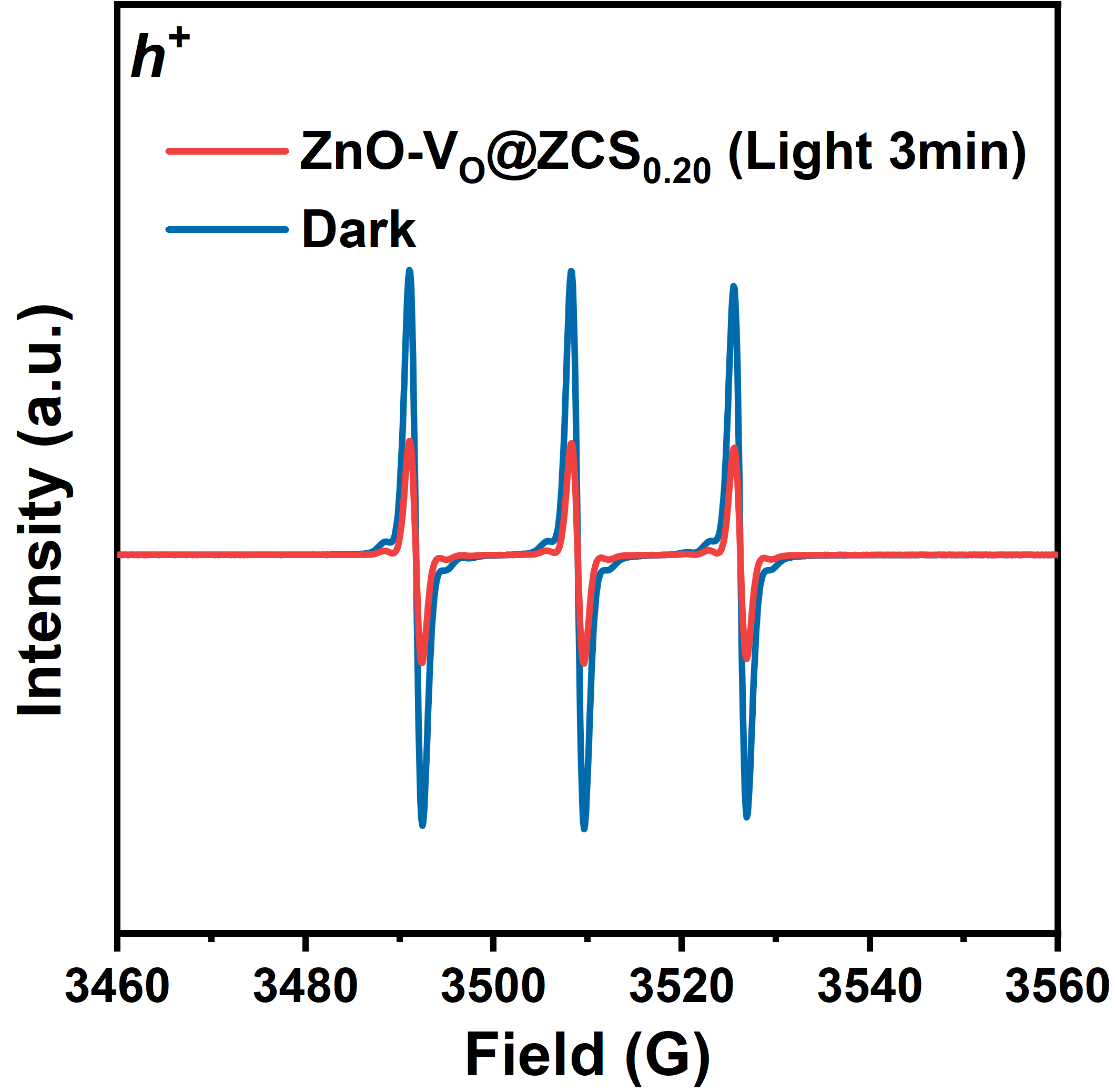


**Fig. S25.**  ESR signals of TEMPO-*h^+^* adducts over ZnO-V_O_@ZCS_0.20_.

[1] P. Samadipakchin, H. R. Mortaheb, A. Zolfaghari, *J. Photoch. Photobio. A* **2017**, 337, 91.

[2] G. Liu, J. Chen, Z. Xie, S. Lin, L. Xie, Y. Deng, C. Lu, *ACS Appl. Energy Mater.* **2022**, 5, 3502.

[3] G. Kresse, J. Furthmüller, *Comput. Mater. Sci.* **1996**, 6, 15.

[4] G. Kresse, J. Furthmüller, *Phys. Rev. B* **1996**, 54, 11169.

[5] J. P. Perdew, K. Burke, M. Ernzerhof, *Phys. Rev. Lett.* **1996**, 77, 3865.

[6] S. Grimme, S. Ehrlich, L. Goerigk, *J. Comput. Chem.* **2011**, 32, 1456.

[7] J. P. Perdew, M. Ernzerhof, K. Burke, *J. Chem. Phys.* **1996**, 105, 9982.

[8] A. V. Krukau, O. A. Vydrov, A. F. Izmaylov, G. E. Scuseria, *J. Chem. Phys.* **2006**, 125, 224106.

[9] L. Franklin, C. E. Ekuma, G. L. Zhao, D. Bagayoko, *J. Phys. Chem. Solids* **2013**, 74, 729.

[10] T. G. Edossa, M. M. Woldemariam, *Adv. Condens. Matter Phys.* **2020**, 2020, 4693654.

[11] B. Khamala, L. Franklin, Y. Malozovsky, A. Stewart, H. Saleem, D. Bagayoko, *Comput. Condens. Matter* **2016**, 6, 18.

[12] Z. Gao, S. Lv, Y. Wang, Z. Xu, Y. Zong, Y. Tao, Y. Zhao, X. Liu, S. Yu, M. Luo, N. Khaorapapong, R. Zhang, Y. Yamauchi, *Adv. Sci.* **2024**, 11, 2406530.

[13] X. Zhong, Q. Ling, Z. Ren, B. Hu, *Appl. Catal. B Environ. Energy* **2023**, 326, 122398.

[14] X. Pei, P. He, K. Yu, Y. Li, Y. Tang, L. Ma, *Adv. Funct. Mater.* **2024**, 34, 2410827.

[15] F. Zhang, H. Dong, Y. Li, D. Fu, L. Yang, Y. Shang, Q. Li, Y. Shao, W. Gang, T. Ding, T. Chen, W. Zhu, *Adv. Sci.* **2024**, 11, 2305439.

[16] W. Wang, Q. Luo, L. Li, Y. Wang, X. Huo, S. Chen, X. Du, N. Wang, *Adv. Funct. Mater.* **2023**, 33, 2302913.

[17] F. He, Q. Xiao, Y. Chen, H. Wang, X. Wang, *Appl. Catal. B Environ. Energy* **2024**, 343, 123525.

[18] Q. Meng, L. Wu, T. Chen, Y. Xiong, T. Duan, X. Wang, *Environ. Sci. Technol.* **2024**, 58, 15333.
